# Changes in Mortality Inequalities in Urban and Rural Populations during 1990–2018: Lithuanian Experience

**DOI:** 10.3390/medicina57080750

**Published:** 2021-07-25

**Authors:** Olga Mesceriakova-Veliuliene, Ramune Kalediene

**Affiliations:** Department of Health Management, Faculty of Public Health, Lithuanian University of Health Sciences, Tilžės St. 18, LT-47181 Kaunas, Lithuania; ramune.kalediene@lsmuni.lt

**Keywords:** mortality, leading causes of death, urban, rural, inequalities, Lithuania

## Abstract

*Background and Objectives*: Reduction in health inequalities is a highly important task in public health policies worldwide. In Lithuania, inequalities in mortality by place of residence are among the greatest, compared to other European Union (EU) countries. However, studies on inequalities in mortality by place of residence over a long-term period have not been investigated in Lithuania. The aim of this study was to present changes in mortality inequalities in urban and rural populations during 1990–2018. *Materials and Methods*: Mortality rates from all causes, cardiovascular diseases, cancer, external causes, and gastrointestinal diseases in urban and rural population by sex were calculated per 100,000 populations and were standardized by age. Inequalities in mortality were assessed using rate differences and rate ratio. For the assessment of inequality trends during 1990–2018, the joinpoint regression analysis was applied. *Results*: Mortality between urban and rural populations varied. In rural areas, mortality lower than that in urban areas was observed only in 1990 among women, in case of mortality from cancer and gastrointestinal diseases (compared with in 2018) (*p* < 0.05). In 2018, mortality from all causes, cardiovascular diseases, and external causes in urban and rural areas was lower than in 1990 in both sexes. However, mortality from gastrointestinal diseases was higher (*p* < 0.05). In 2018, mortality from cancer among both sexes was lower only in urban areas (*p* < 0.05). Mortality inequalities between rural and urban areas decreased statistically significantly only among men from external causes and from all causes (respectively, on average, by 0.52% per year and, on average, by 0.21% per year). Meanwhile, mortality from cardiovascular and gastrointestinal diseases increased in both sexes, and mortality from cancer and all causes of death increased among women. The increase in the inequalities of mortality from gastrointestinal diseases was the most rapid: among men—on average, by 0.69% per year, and among women—on average, by 1.43% per year, *p* < 0.0001. *Conclusions*: During 1990–2018, the inequalities in mortality by place of residence in Lithuania statistically significantly decreased only among men, in terms of mortality from external causes and from all causes. Therefore, reduction in inequalities in mortality must be the main the health policy challenge in Lithuania.

## 1. Introduction

Compared to other countries of the EU, Lithuania is distinguished not only by a very high overall mortality of the population [1], but also by very large inequalities in mortality by socio-demographic groups [2,3]. However, territorial inequalities in mortality in Lithuania are of the greatest—Lithuanian rural areas have significantly higher men and women mortality rates than urban areas do, resulting in shorter mean life expectancy [4].

Territorial inequalities in mortality in Lithuania indirectly reflect the greater social and psychological stress experienced by the rural population related to the economic, social, and cultural factors that adversely affect health [5]; in addition, they also reflect health inequalities, as access to high-quality preventive, outpatient, and inpatient services for rural residents is more complicated and takes longer than it does for urban residents [6,7]. This is related to the unequal distribution of healthcare professionals [8], shortcomings in the organization and management of the efficiency and timeliness of healthcare services, and longer distances to healthcare institutions [9,10]. The poorer health of the rural population is also caused by their lack of conscious care for their own health [7,11,12]. Due to the social and material situation of the low-income population, their access to healthcare services and full participation in public life are insufficient [13]. This is especially relevant in Lithuanian villages that are remote from major cities [4].

The large and growing inequalities in the mortality of the population show [14,15,16,17] that Lithuania is failing to achieve the goals set out by the World Health Organization—i.e., to reduce social and health inequalities [18]. Therefore, continuous monitoring of the population’s mortality and inequalities is essential to reduce them. Lithuania, along with other countries, pays a great deal of attention to the reduction in health inequalities. The main goal of the Lithuanian Health Strategy 2014–2025 is the attainment of improved health of the Lithuanian population, as well as reduced mortality rates and increased life expectancy [19]. This goal is supposed to be achieved by building a safer social environment, reducing health inequalities and social deprivation, fostering healthy lifestyles of the population and a culture of promoting it, creating a working and living environment favorable for health, and ensuring a high-quality and more effective healthcare oriented towards the population’s needs. Since 2014, even greater attention has been paid to reducing health inequalities in Lithuania with the Action Plan for Reducing Health Inequalities being implemented [20]. It aims to reduce the differences in health and access to healthcare across regions and social groups.

Even though studies of such a type have already been conducted in Lithuania before, mortality inequalities were analyzed in a shorter time period and in other age groups [21,22,23]. Due to significant health inequalities in Lithuania, a decision to analyze inequalities in mortality from the leading causes of death by place of residence within a long period was made. Such analysis has not been performed before. The aim of this study was to present the changes in mortality inequalities in urban and rural populations during 1990–2018.

## 2. Materials and Methods

### 2.1. Data Sources

Information on deaths among men and women in urban and rural was obtained from the State Register of Death Cases and Their Causes. The size of the average annual population during 1990–2018 was obtained from the Database of Indicators of Statistics Lithuania [7].

### 2.2. Population

In Lithuania, 1,216,149 people died during the analyzed period (668,319 died from cardiovascular diseases, 226,878—from cancer, 137,808—from external causes (accidents; intentional self-harm; accidental drowning and submersion; accidental poisoning by and exposure to noxious substances; assault, and others) and 48,930 died from gastrointestinal diseases (gastric, duodenal, and peptic ulcer; diseases of appendix; alcoholic liver disease; fibrosis and cirrhosis of liver, and others)). During the studied period, population size dropped from 3.7 to 2.8 million people (in urban areas, from 2.5 to 1.9 million people, and in rural areas, from 1.2 to 0.9 million people). More detailed information about population and deaths from all causes and from major causes during 1990–2018 by place of residence and sex is presented in the Supplementary Material (see Tables S1–S3).

The data were analyzed by the place of residence and sex. Urban and rural populations were categorized on the basis of the Lithuanian classification supplied by Statistics Lithuania, the categorization being as follows: (1) urban population refers to those persons who live in cities and towns, i.e., in the population areas with closely built permanent dwellings and with the resident population of more than 3000; (2) rural population refers to those persons who live in the population areas without any signs of a town or a city (villages, small towns, and steadings) [24].

### 2.3. Statistical Analysis

The analysis for this study included all-cause mortality (age 0+), as well as mortality from four leading causes of death in Lithuania. The analyzed causes of death and the corresponding codes of the International Classification of Diseases are presented in Table 1.

Mortality rates from the analyzed causes were calculated by the place of residence (urban or rural) among men and women per 100,000 population. Mortality rates were age-standardized using the European Standard Population (1976) as recommended by the WHO.

Changes in the magnitude of mortality inequalities by place of residence were assessed using the easily interpretable measure of absolute (rate difference of mortality = rural–urban) and relative (rate ratio of mortality = rural/urban) terms with its 95% confidence interval. For the assessment of inequality trends during 1990–2018, the joinpoint regression analysis was applied. In this analysis, the best fitting points where the rate changes significantly, increase or decrease, were chosen [25]. The analysis started with a minimum number of cut-off points, testing whether one or more cut-off points were statistically significant and whether or not they could be added to the model. In the final model, each joinpoint indicated a statistically significant change in a trend; computed next was the annual percent of change for each of those trends. For the joinpoint analysis, the overall level of significance was set at *p* = 0.05. Significant changes included changes in the direction or rate of the trend. The permutation test—testing the number of joinpoints 0 against 3—was applied in this case because the 29-year period did not allow for obtaining statistically significant results for more joinpoints. Coefficients of regression multiplied by 100 were presented as average annual changes (AAC), which were considered to be statistically significant at *p* < 0.05.

## 3. Results

The age-standardized mortality from all causes and from the leading causes of death in urban and rural areas among men and women in 1990 and 2018 along with the changes in mortality are presented in Table 2. Mortality between urban and rural population varied. In 1990, among men and women, mortality from all causes and from external causes was higher in rural than in urban areas, as was the case with mortality from cardiovascular diseases among women (*p* < 0.05). Lower mortality was observed only among women—from cancer and from gastrointestinal diseases (*p* < 0.05). In 2018, mortality from cardiovascular diseases and from all causes in both sexes and from external causes among men was higher in rural areas than it was in urban areas (*p* < 0.05).

The analysis of data for the years 1990 and 2018 showed that in 2018, mortality from all causes, cardiovascular diseases, and external causes in urban and rural areas was lower than in 1990 in both sexes. However, mortality from gastrointestinal diseases was higher (*p* < 0.05). In 2018, mortality from cancer among both sexes was lower only in urban areas (*p* < 0.05). 

The study also showed that among men, mortality from all the analyzed causes of death was higher than that among women (*p* < 0.05). More detailed information for the period of 1990–2018 concerning mortality by place of residence among men and women with confidence intervals for each year and trends are presented in the Supplementary Material (see Tables S4–S7).

Differences in the age-standardized mortality rate and rate ratio between the rural and urban areas in 1990 and in 2018, as well as changes in absolute and relative inequalities in mortality between 1990 and 2018 are presented in Table 3.

The most prominent differences between rural and urban areas among men and women were found in rates of all-cause mortality in 1990 (among men, 200.18/100,000 population; among women, 88.50/100,000 population) and in 2018 (among men, 93.68/100,000 population; among women, 81.91/100,000 population). The smallest differences among men were found in mortality from cancer (−4.81/100,000 population in 1990 and −0.50/100,000 population in 2018), while among women, the smallest difference was observed in mortality from gastrointestinal diseases (−6.40/100,000 population in 1990 and −1.10/100,000 population in 2018).

The analysis of the rate ratio showed that differences between the rate ratio in 1990 and in 2018 were not statistically significant. In 2018, compared to 1990, a higher rate ratio was found in mortality from cardiovascular diseases, cancer, and gastrointestinal diseases among both sexes and from all causes among women (*p* > 0.05).

More detailed information on rate differences and rate ratio by place of residence among men and women for each year are presented in the Supplementary Material (see Tables S8 and S9).

For the assessment of trends in the rate ratio of mortality from major causes by place of residence (rural/urban) and sex during 1990–2018, the joinpoint regression analysis was performed. Rate ratio of all-cause mortality in both sexes varied unevenly: there were 1 or 3 statistically significant cut-off points, except for mortality from cancer among women and mortality from gastrointestinal diseases among men (Table 4).

Three statistically significant cut-off points were found in trends of the rate ratio of mortality from cardiovascular diseases, external causes, and all causes in both sexes and the rate ratio of mortality from gastrointestinal diseases among women. Among men, one statistically significant cut-off point was found in the rate ratio of mortality from cancer. However, the changes in the rate ratio of mortality were statistically significant not during all the periods between the cut-off points.

The rate ratio of mortality from all causes decreased statistically significantly in both sexes (among men, from 1.24 in 2004 to 1.08 in 2018 (on average, by 0.86% per year); among women, from 1.26 in 2005 to 1.16 in 2018 (on average, by 0.54% per year)). Among men, a statistically significant decrease in the rate ratio of mortality from cardiovascular diseases (from 1.25 in 2003 to 1.12 in 2018 (on average, by 0.37% per year)) and from external causes (from 1.53 in 2008 to 1.33 in 2018 (on average, by 1.68% per year)) was observed.

There was an increase in the rate ratio of mortality from all causes in both sexes (among men, form 1.15 in 1990 to 1.25 in 1998 (on average, by 0.91% per year), and among women, from 1.12 in 1990 to 1.19 in1998 (on average, by 0.85% per year)). Among men, an increase was observed in the rate ratio of mortality from cardiovascular diseases (from 1.05 in 1990 to 1.20 in 1996 (on average, by 1.69% per year)) and from cancer (from 0.98 in 1990 to 1.10 in 1998 (on average, by 1.22% per year)). Among women, there was an increase in the rate ratio of mortality from external causes (form 1.22 in 1993 to 1.69 in 2007 (on average, by 1.88% per year)) and from gastrointestinal diseases (form 0.81 in 2000 to 1.11 in 2006 (on average, by 6.73% per year)).

The analysis of the influence of the differences in mortality from the leading causes of death in men and women between the rural and urban population on the differences in all-cause mortality showed that in 1990 and 2018, in women, the differences in all-cause mortality were most influenced by differences in mortality from cardiovascular diseases (93.45% in 1990 and 86.72% in 2018), while in men, from external causes (48.63% in 1990) and from cardiovascular diseases (63.78% in 2018) (Table 5). Meanwhile, during the same period, differences in mortality from cancer had the smallest effect among women (−26.05% in 1990 and −3.50% in 2018), and differences in mortality from gastrointestinal diseases (−2.61% in 1990) and cancer (−0.53% in 2018)—among men.

## 4. Discussion

This study is one of the largest in Lithuania covering all-cause mortality and mortality from the four leading causes of death, as well as inequalities in mortality between rural and urban areas over a 29-year period (i.e., mortality and its inequalities were analyzed since the beginning of Lithuania’s independence (11 March 1990)). For many years, the structure of the causes of death of the Lithuanian population has been dominated by three main causes of death, which have remained unchanged—cardiovascular diseases, cancer, and external causes of death. In 2019, they accounted for 81.9% of all causes of death [4]. Such structure of the causes of death was once common in many economically developed countries [26], yet recently, it is characteristic only of Croatia, Slovenia, Latvia, Slovakia, and Estonia [27]. Since 2004, gastrointestinal diseases (5% of all deaths) have taken the fourth place in the structure of the causes of death in Lithuania, replacing diseases of the respiratory system that had occupied this place for a long time [28]. However, according to other authors, the structure of the causes of death in Lithuania is inaccurate and may change slightly after correcting errors in filling out the medical death certificates [29]. An incorrectly identified cause of death or other errors in completing a death certificate artificially increase the significance of certain causes of death (particularly, cardiovascular diseases) in the structure of the causes of death. In Lithuania, as in other countries, ischemic heart disease is over-diagnosed most frequently [30,31,32].

The restoration of Lithuania’s independence marked the beginning of intensive internal migration, democratic reforms, the economic reform, and other urgent work of building state institutions and structures, which, for some people, meant the loss of a stable social status, unemployment, growing social inequality and, at the same time, tensions in society [7,19,33]. In almost all post-communist countries, sudden socio-economic changes led to a health crisis that had a negative impact on the health of society as a whole. According to the data of our study, after the restoration of Lithuania’s independence, there was an increase in all-cause mortality and mortality from the main causes of death in both urban and rural areas, the increase in mortality from external causes and diseases of the digestive system being especially significant. According to the data of Statistics Lithuania, the mean life expectancy was decreasing during that period [7].

As the EU economy growth was slowing down, the Lithuanian economy and living cost continued to grow rapidly [27]. Due to the continuing living costs growth, income inequalities and poverty remain a challenge for Lithuania, which increases the risk of social exclusion and policy uncertainty in the country [27,34]. Even though measures have been taken to reduce poverty, social protection expenditure in Lithuania remains one of the lowest in the EU, and the level of the risk of poverty or social exclusion (28.3%) remains one of the highest in the EU (the EU mean being 21.7%). In Lithuania, the highest at-risk-of-poverty rate is observed among those over 65 years of age (31.6%), and income inequality, even on the day people receive retirement pensions or social benefits, remains one of the highest in the EU. The income of the richest 20% of Lithuanian households was about 7 times higher than that of the poorest 20% of households [27].

However, during the analyzed period, some positive changes were observed in Lithuania as —namely, a decrease in unemployment [7], an increase in the mean size of retirement pensions (up to EUR 413) [7], an improvement in living conditions [35,36] and the cost of living [27], and an improvement in healthcare due to increased funding [6]. These factors led to improved health and declining mortality of the population. According to the results of our study, in 1990–2018, mortality from the analyzed causes of death in urban and rural areas changed unevenly; three cut-off points were found (except for mortality from cancer), and positive and negative mortality trends were observed between the cut-off points. However, the analysis of changes in mortality during the whole analyzed period (1990–2018) revealed positive changes in Lithuanian urban and rural areas, as in other EU countries: mortality was decreasing, except for mortality from gastrointestinal diseases (mortality from these diseases was increasing) [1]. According to the results of other studies, mortality from this cause of death was also increasing in different education level groups [14].

Differences in mortality and its trends between urban and rural areas led to an increase in the inequality in all-cause mortality and mortality from cardiovascular diseases, cancer, and gastrointestinal diseases among women. The study showed that the increase in the inequalities in all-cause mortality and mortality from cardiovascular diseases and cancer among women was due to a faster reduction in mortality in urban than in rural areas, while the increase in mortality from gastrointestinal diseases was due to a faster increase in mortality in rural areas. The slower decline in mortality in rural areas was due to a number of negative socio-economic factors. According to Statistics Lithuania, the at-risk-of-poverty rate in rural areas is significantly higher than in cities. In 2019, as much as 27.9% of the rural population were at risk of poverty (compared to 17% in urban areas). In addition, the rate of unemployment in rural areas was higher (8.5% compared to 5.3% in urban areas), disposable household income was by 38.5% lower than in urban areas, and as much as 5.7% of the rural population faced severe material deprivation. The quality of housing between urban and rural areas differed especially significantly. Although only 3% of people in urban areas lived in dwellings without a water closet, in rural areas, the percentage of such people reached 24%. The percentages of people living without a bath or shower in urban and rural areas were 4% and 21%, respectively. Moreover, as much as 57% of the rural population did not have a possibility to spend at least a week off outside their home, and 57% would not be able to pay for unforeseen expenses from their own funds. In addition, the rural population had a poorer lifestyle, consumed more alcohol, lemonade or Coca-Cola, sweets, and more frequently smoked and were exposed to smoke; consumed fewer fresh vegetables, and fruit and berries. A smaller proportion of the rural population consulted a family doctor or specialist about health problems, and this population was less likely to participate in prevention programs [7,11,12]. These factors resulted in poorer health in the rural population, leading to higher mortality, a slower decline in mortality (but not from all causes of death), and shorter mean life expectancy [4].

In Lithuania, many measures have been implemented to reduce poverty and social exclusion, including an increase in child benefits, retirement pensions, and minimum monthly wages, the initiation of the indexation of basic social benefits, longer payments of the unemployment social insurance benefit, increased social support for students, targeted compensation for people in need of permanent care or nursing care, the initiation of benefits for multiple births and benefits for the care of a child when the parent is learning or studying, and the approval of the basic package of services for the family [37].

However, the results of our study indicate that not all aims related to the reduction in health and social inequalities listed in the first Lithuanian Health Program and the WHO political documents have been achieved in Lithuania [18,38]. In most EU countries, the reduction in health inequalities among different socio-demographic/socio-economic groups have already been one of the most important directions of health policies for several decades. Meanwhile, in Lithuania, considerable efforts in reducing inequalities in health were initiated only in 2014 [19,20]. Therefore, it can be expected that the implementation of the Lithuanian Health Strategy and the Action Plan for Reducing Health Inequalities will help to reduce growing health inequalities.

## 5. Conclusions

An analysis of standardized mortality for men and women in urban and rural areas in 1990 and 2018 showed that mortality in the rural areas was lower only in 1990 among women in case of mortality from cancer and gastrointestinal diseases (*p* < 0.05). In 2018, mortality from all causes, cardiovascular diseases, and external causes in urban and rural areas was lower than in 1990 in both sexes. However, mortality from gastrointestinal diseases was higher (*p* < 0.05). In 2018, mortality from cancer among both sexes was lower only in urban areas (*p* < 0.05) The joinpoint regression analysis showed that during 1990–2018, inequalities in mortality (rate ratio) of men and women varied unevenly, but the analysis of the whole period (1990–2018) showed that in both sexes, there was an increase in inequalities in mortality from cardiovascular diseases and gastrointestinal diseases, and among women—from cancer and all causes, while a decrease in inequalities was observed in mortality from external causes and all causes among men (*p* < 0.05). 

Summing up, during 1990–2018, mortality of the residents of Lithuania remained high, decreased (except gastrointestinal diseases) and changed very unevenly. Mortality was also greater in rural areas than in urban areas, although rural/urban inequalities tended to increase. This proves the great importance of not only further improvement of the quality of healthcare services, but also significant strengthening of the prevention of diseases, early diagnostics, and health promotion in Lithuanian population (paying special attention to the rural population), which could undoubtedly reduce mortality and its inequalities in the country.

## Figures and Tables

**Table 1:** Causes of death and the corresponding codes of the International Classification of Disease (ICD).

Causes of Death	Short ICD-9 (1990–1992)	ICD-9 (1993–1997)	ICD-10 (From 1998)
Cardiovascular diseases	84–102	390–459	I00–I99
Cancer	45–66	140–209	C00–C97
External causes	160–175	E800–E999	V01–Y98
Gastrointestinal diseases	115–127	520–579	K00–K93
All causes	1–185	001–999	A00–Y98

**Table 2:** Age-standardized mortality rates (ASMR) per 100,000 population by place of residence and sex in 1990 and 2018, and rate differences in mortality change between rural and urban areas.

Causes of Death	Sex	1990		2018		Changes ^a^ (100,000 population)
		Urban ASMR (95% CI)	Rural ASMR (95% CI)	Urban ASMR (95% CI)	Rural ASMR (95% CI)	
Cardiovascular diseases	Men	720.32 (700.08; 740.56)	758.33 (736.63; 780.03)	498.80 (485.54; 512.06)	558.66 * (539.72; 577.6)	21.85
	Women	446.25 (435.15; 457.35)	528.95* (515.57; 542.33)	267.77 (261.35; 274.19)	338.8 * (328.42; 349.18)	−11.67
Cancer	Men	288.4 (275.84; 300.96)	283.59 (269.45; 297.73)	264.34 (254.32; 274.36)	263.84 (250.46; 277.22)	4.31
	Women	149.46 (142.54; 156.38)	126.41* (118.13; 134.69)	125.92 (120.32; 131.52)	123.05 (114.51; 131.59)	20.21
External causes	Men	178.67 (170.15; 187.19)	276.01 * (261.55; 290.47)	114.44 (107.38; 121.5)	152.03 * (140.99; 163.07)	−59.75
	Women	44.02 (40.34; 47.7)	64.22* (57.58; 70.86)	28.92 (25.88; 31.96)	36.90 (31.56; 42.24)	−12.22
Gastrointestinal diseases	Men	32.49 (28.39; 36.59)	27.27 (22.81; 31.73)	64.32 (59.2; 69.44)	68.68 (61.42; 75.94)	9.58
	Women	21.51 (18.91; 24.11)	15.11 * (12.33; 17.89)	30.19 (27.37; 33.01)	29.09 (25.09; 33.09)	5.30
All causes	Men	1367.07 (1340.49; 1393.65)	1567.25 * (1535.25; 1599.25)	1112.12 (1091.94; 1132.3)	1205.8* (1177.48; 1234.12)	−106.50
	Women	740.25 (725.67; 754.83)	828.75 * (810.17; 847.33)	526.06 (515.7; 536.42)	607.97 * (591.51; 624.43)	−6.59

CI, confidence intervals. * *p* < 0.05 compared to urban areas. ^a^ change in the difference in mortality between the rural and urban areas was calculated: (ASMR_2018_, _rural_—ASMR_1990_, _rural_)—(ASMR_2018, urban_—ASMR_1990, urban_).

**Table 3:** Differences in age-standardized mortality rate and rate ratio between rural and urban populations in 1990 and 2018, and changes in absolute (RD) and relative (RR) inequalities in mortality between 1990 and 2018.

Causes of Death	Sex	1990		2018		Changes (%)	
		RD (95% CI)	RR (95% CI)	RD (95% CI)	RR (95% CI)	RD ^a^	RR ^b^
Cardiovascular diseases	Men	38.01 (36.55; 39.47)	1.05 (0.98; 1.13)	59.86 * (54.18; 65.54)	1.12 (1.03; 1.22)	57.48	58.33
	Women	82.70 (80.42; 84.98)	1.19 (1.09; 1.29)	71.03 * (67.07; 74.99)	1.27 (1.13; 1.41)	−14.11	29.63
Cancer	Men	−4.81 (−6.39; -3.23)	0.98 (0.87; 1.10)	−0.50 (−3.86; 2.86)	0.99 (0.88; 1.12)	−89.61	−100.00
	Women	−23.05 (−24.41; -21.69)	0.85 (0.70; 1.00)	−2.87 * (−5.81; 0.07)	0.98 (0.81; 1.17)	−87.55	−650.00
External causes	Men	97.34 (91.4; 103.28)	1.54 (1.37; 1.74)	37.59 * (33.61; 41.57)	1.33 (1.13; 1.56)	−61.38	−63.64
	Women	20.20 (17.24; 23.16)	1.46 (1.12; 1.86)	7.98 * (5.68; 10.28)	1.28 (0.89; 1.74)	−60.50	−64.29
Gastrointestinal diseases	Men	−5.22 (−5.58; -4.86)	0.84 (0.55; 1.21)	4.36 * (2.22; 6.50)	1.07 (0.83; 1.35)	−183.53	328.57
	Women	−6.40 (−6.58; −6.22)	0.70 (0.39; 1.15)	−1.10 * (−2.28; 0.08)	0.96 (0.64; 1.38)	−82.81	−650.00
All causes	Men	200.18 (194.76; 205.6)	1.15 (1.09; 1.20)	93.68 * (85.54; 101.82)	1.08 (1.02; 1.15)	−53.20	−87.50
	Women	88.50 (84.50; 92.50)	1.12 (1.04; 1.20)	81.91 (75.81; 88.01)	1.16 (1.07; 1.25)	−7.45	25.00

CI, confidence intervals. RD, rate differences (rural − urban)/100,000 population. RR, rate ratio (rural/urban)/times. * *p* < 0.05 compared to the year 1990. ^a^ change in absolute inequalities calculated: 100 × (RD_2018_ − RD_1990_)÷RD_1990_. ^b^ change in relative inequalities calculated: 100×(RR_2018_ − RR_1990_)÷(RR_2018_ − 1).

**Table 4:** Trends in rate ratio (rural/urban population) of mortality by place of residence and sex in 1990–2018.

Causes of Deaths	Sex	Number of Cut-Off Points	Years of Cut-Off Point	Period 1		Period 2		Period 3		Period 4		Whole Period (1990–2018)
				Years	ACC (95% CI), p	Years	ACC (95% CI), *p*	Years	ACC (95% CI), *p*	Years	ACC (95% CI), *p*	ACC (95% CI), *p*
Cardiovascular diseases	Men	3	1996 2000 2003	1990–1996	1.69 (0.54; 2.86), 0.00593	1996–2000	−0.51 (−3.80; 2.91), 0.75762	2000–2003	1.59 (−5.03; 8.68), 0.63030	2003–2018	−0.37 (−0.65; −0.09), 0.01297	0.16 (0.005; 0.317), 0.04395
	Women	3	1994 1997 2009	1990–1994	−0.75 (−3.41; 1.98), 0.56877	1994–1997	3.08 (−5.39; 12.30), 0.47025	1997–2009	0.43 (−0.14; 1.02), 0.13361	2009–2018	−0.51 (−1.29; 0.27), 0.18803	0.38 (0.208; 0.560), 0.00012
Cancer	Men	1	1998	1990–1998	1.22 (0.05; 2.41), 0.04087	1998–2018	−0.18 (−0.47; 0.11), 0.21061	-	-	-	-	0.11 (0.079; 0.293), 0.247
	Women	0	-	-	-	-	-	-	-	-	-	0.48 (0.292; 0.664), 0.00001
External causes	Men	3	1993 1999 2008	1990–1993	−4.28 (−10.09; 1.91), 0.16147	1993–1999	2.09 (−0.73; 4.99), 0.13979	1999–2008	−0.63 (−1.98; 0.73), 0.34464	2008–2018	−1.68 (−2.63; −0.71), 0.00165	−0.52 (−0.807; −0.240), 0.00078
	Women	3	1993 2007 2013	1990–1993	−5.24 (−15.49; 6.25), 0.33892	1993–2007	1.88 (0.67; 3.11), 0.00403	2007–2013	−2.21 (−7.09; 2.93), 0.37414	2013–2018	−3.90 (−8.70; 1.15), 0.12083	−0.007 (−0.502; 0.490), 0.976
Gastrointestinal diseases	Men	3	1997 2000 2006	1990–1997	3.14 (−0.31; 6.71), 0.07293	1997–2000	−5.91 (−27.05; 21.37), 0.62417	2000–2006	6.73 (0.83; 12.99), 0.02683	2006–2018	−0.68 (−2.16; 0.83), 0.35923	0.69 (0.383; 0.997), 0.00008
	Women	0	-	-	-	-	-	-	-	-	-	1.43 (0.961; 1.906), 0.00000
All causes	Men	3	1998 2001 2004	1990–1998	0.91 (0.32; 1.49), 0.00393	1998–2001	−0.80 (−5.94; 4.61), 0.75571	2001–2004	0.94 (−4.29; 6.44), 0.71874	2004–2018	−0.86 (−1.11; −0.61), 0.00000	−0.21 (−0.369; −0.059), 0.009
	Women	3	1998 2001 2005	1990–1998	0.85 (0.18; 1.53), 0.01575	1998–2001	−1.05 (−6.95; 5.22), 0.72488	2001–2005	1.85 (-1.23; 5.03), 0.22785	2005–2018	−0.54 (−0.86; −0.22), 0.00229	0.16 (0.027; 0.299), 0.020

ACC, average annual change; CI, confidence interval.

**Table 5:** Differences in men and women mortality from the leading causes of death (RD) per 100,000 population between rural and urban population and their impact (%) on differences in all-cause mortality.

Causs of Death	Sex	1990		2018	
		RD	% ^a^	RD	% ^a^
Cardiovascular diseases	Men	38.01	18.99	59.86	63.78
	Women	82.70	93.45	71.03	86.72
Cancer	Men	−4.81	−2.40	−0.50	−0.53
	Women	−23.05	−26.05	−2.87	−3.50
External causes	Men	97.34	48.63	37.59	40.05
	Women	20.20	22.82	7.98	9.74
Gastrointestinal diseases	Men	−5.22	−2.61	4.36	4.65
	Women	−6.40	−7.23	−1.10	−1.34
All causes	Men	200.18	100	93.68	100
	Women	88.50	100	81.68	100

RD, rate differences (rural/urban)/100,000 population. ^a^ the effect of differences in mortality from the leading causes of death on differences in all-cause mortality was calculated: (RD_cause of death_ × 100)/RD_all causes._

## Data Availability

Data can be made available from the corresponding author on reasonable request.

## Supplementary Materials

The following are available online at https://www.mdpi.com/article/10.3390/medicina57080750/s1, Table S1: Table S1. Average annual number of men and women in urban and rural areas during 1990–2018, Table S2: Number of deaths among men in urban and rural areas during 1990–2018, Table S3: Number of deaths among women in urban and rural areas during 1990–2018, Table S4: Age-standardized mortality rates from all causes and major causes among men in 1990–2018 (100,000 population), Table S5: Age-standardized mortality rates from all causes and major causes among women in 1990–2018 (100,000 population), Table S6: Trends in age-standardized mortality rates among men by place of residence during 1990–2018, Table S7: Trends in age-standardized mortality rates among women by place of residence during 1990–2018, Table S8: Age-standardized mortality rate differences (rural-urban) among men and women during 1990–2018 (100,000 population), Table S9: Age-standardized mortality rate ratio (rural/urban) among men and women during 1990–2018.
