# Supplementary material for: Changes in Mortality Inequalities in Urban and Rural Populations during 1990–2018: Lithuanian Experience
Source: Medicina (Kaunas). 2021 Jul 25;57(8):750. doi: 10.3390/medicina57080750 (PMC8398674; doi:10.3390/medicina57080750)
Supplement: Supplementary file 1 [file medicina-57-00750-s001.zip › medicina-1283507-supplementary.pdf]

Supplementary material

Table S1. Average annual number of men and women in urban and rural areas during 1990-2018.

Years	Men				Women				All				All
	Urban		Rural		Urban		Rural		Men		Women		
	N	%	N	%	N	%	N	%	N	%	N	%	
1990	1,186,550	32.09	562,674	15.22	1,333,779	36.07	614,835	16.63	1,749,224	47.30	1,948,614	52.70	3,697,838
1991	1,190,086	32.13	561,783	15.17	1,339,013	36.15	613,252	16.56	1,751,869	47.29	1,952,265	52.71	3,704,134
1992	1,186,067	32.05	563,316	15.22	1,334,858	36.08	615,873	16.64	1,749,383	47.28	1,950,731	52.72	3,700,114
1993	1,174,005	31.88	565,684	15.36	1,324,410	35.96	618,514	16.80	1,739,689	47.24	1,942,924	52.76	3,682,613
1994	1,158,894	31.69	566,396	15.49	1,313,393	35.91	618,461	16.91	1,725,290	47.18	1,931,854	52.82	3,657,144
1995	1,142,822	31.49	566,565	15.61	1,302,710	35.90	617,005	17.00	1,709,387	47.10	1,919,715	52.90	3,629,102
1996	1,132,093	31.43	561,610	15.59	1,298,685	36.06	609,225	16.92	1,693,703	47.03	1,907,910	52.97	3,601,613
1997	1,120,359	31.34	558,389	15.62	1,293,219	36.17	603,170	16.87	1,678,748	46.96	1,896,389	53.04	3,575,137
1998	1,105,127	31.14	559,480	15.76	1,282,726	36.14	601,998	16.96	1,664,607	46.90	1,884,724	53.10	3,549,331
1999	1,092,628	31.00	558,303	15.84	1,274,518	36.16	598,789	16.99	1,650,931	46.85	1,873,307	53.15	3,524,238
2000	1,079,945	30.86	557,671	15.94	1,265,695	36.17	596,225	17.04	1,637,616	46.80	1,861,920	53.20	3,499,536
2001	1,066,774	30.74	555,468	16.00	1,255,807	36.18	592,769	17.08	1,622,242	46.74	1,848,576	53.26	3,470,818
2002	1,055,835	30.67	551,491	16.02	1,247,188	36.22	588,553	17.09	1,607,326	46.68	1,835,741	53.32	3,443,067
2003	1,043,523	30.56	549,035	16.08	1,236,462	36.20	586,193	17.16	1,592,558	46.63	1,822,655	53.37	3,415,213
2004	1,027,547	30.43	545,594	16.16	1,221,661	36.18	582,273	17.24	1,573,141	46.58	1,803,934	53.42	3,377,075
2005	1,008,471	30.35	536,893	16.16	1,203,618	36.23	573,546	17.26	1,545,364	46.51	1,777,164	53.49	3,322,528
2006	990,840	30.30	526,789	16.11	1,188,167	36.34	564,113	17.25	1,517,629	46.41	1,752,280	53.59	3,269,909
2007	977,988	30.27	519,119	16.07	1,177,883	36.45	556,304	17.22	1,497,107	46.33	1,734,187	53.67	3,231,294
2008	966,638	30.22	513,748	16.06	1,168,270	36.53	549,575	17.18	1,480,386	46.29	1,717,845	53.71	3,198,231
2009	953,885	30.16	507,891	16.06	1,158,363	36.62	542,777	17.16	1,461,776	46.22	1,701,140	53.78	3,162,916
2010	931,128	30.06	497,583	16.07	1,136,967	36.71	531,604	17.16	1,428,711	46.13	1,668,571	53.87	3,097,282
2011	907,995	29.99	487,372	16.09	1,113,370	36.77	519,378	17.15	1,395,367	46.08	1,632,748	53.92	3,028,115
2012	896,102	29.99	480,099	16.07	1,101,334	36.86	510,238	17.08	1,376,201	46.06	1,611,572	53.94	2,987,773
2013	888,578	30.04	473,865	16.02	1,093,346	36.97	501,900	16.97	1,362,443	46.06	1,595,246	53.94	2,957,689
2014	881,978	30.08	469,148	16.00	1,086,618	37.06	494,623	16.87	1,351,126	46.08	1,581,241	53.92	2,932,367
2015	873,571	30.07	464,361	15.99	1,079,349	37.16	487,629	16.79	1,337,932	46.06	1,566,978	53.94	2,904,910
2016	860,905	30.02	459,992	16.04	1,066,243	37.17	481,091	16.77	1,320,897	46.05	1,547,334	53.95	2,868,231
2017	848,512	30.00	456,228	16.13	1,049,383	37.10	474,280	16.77	1,304,740	46.13	1,523,663	53.87	2,828,403
2018	842,614	30.08	453,828	16.20	1,037,432	37.03	467,669	16.69	1,296,442	46.28	1,505,101	53.72	2,801,543

Table S2. Number of deaths among men in urban and rural areas during 1990-2018.

Years	Cardiovascular diseases				Cancer				External causes				Gastrointestinal diseases				Others				All causes				All
	Urban		Rural		Urban		Rural		Urban		Rural		Urban		Rural		Urban		Rural		Urban		Rural		
	N	%	N	%	N	%	N	%	N	%	N	%	N	%	N	%	N	%	N	%	N	%	N	%	
1990	5196	25.25	4952	24.06	2275	11.06	1667	8.10	1926	9.36	1494	7.26	276	1.34	161	0.78	1281	6.23	1350	6.56	10954	53.23	9624	46.77	20578
1991	5131	23.63	4970	22.89	2340	10.78	1655	7.62	2325	10.71	1794	8.26	343	1.58	179	0.82	1552	7.15	1425	6.56	11691	53.84	10023	46.16	21714
1992	5260	23.73	4903	22.12	2357	10.63	1849	8.34	2326	10.49	1744	7.87	381	1.72	189	0.85	1626	7.34	1529	6.90	11950	53.92	10214	46.08	22164
1993	5961	24.04	5526	22.29	2418	9.75	1801	7.26	2971	11.98	1967	7.93	377	1.52	226	0.91	1763	7.11	1782	7.19	13490	54.41	11302	45.59	24792
1994	5978	23.60	5207	20.55	2454	9.69	1765	6.97	3205	12.65	2282	9.01	437	1.72	217	0.86	2014	7.95	1775	7.01	14088	55.61	11246	44.39	25334
1995	5528	22.76	5114	21.06	2522	10.39	1788	7.36	3011	12.40	2154	8.87	469	1.93	224	0.92	1985	8.17	1488	6.13	13515	55.66	10768	44.34	24283
1996	5378	23.54	5002	21.89	2469	10.81	1783	7.80	2551	11.17	1955	8.56	412	1.80	224	0.98	1722	7.54	1352	5.92	12532	54.85	10316	45.15	22848
1997	5206	24.10	4538	21.01	2454	11.36	1751	8.11	2403	11.12	1865	8.63	429	1.99	225	1.04	1496	6.93	1235	5.72	11988	55.49	9614	44.51	21602
1998	5067	23.91	4351	20.53	2471	11.66	1788	8.44	2306	10.88	1885	8.89	477	2.25	263	1.24	1350	6.37	1235	5.83	11671	55.07	9522	44.93	21193
1999	5169	24.65	4383	20.90	2505	11.94	1856	8.85	2175	10.37	1916	9.14	452	2.16	225	1.07	1188	5.66	1103	5.26	11489	54.78	9483	45.22	20972
2000	5010	24.55	4067	19.93	2566	12.57	1754	8.59	2241	10.98	1704	8.35	486	2.38	217	1.06	1262	6.18	1101	5.39	11565	56.67	8843	43.33	20408
2001	5363	24.86	4315	20.00	2611	12.10	1753	8.13	2416	11.20	1914	8.87	546	2.53	273	1.27	1262	5.85	1118	5.18	12198	56.55	9373	43.45	21571
2002	5518	25.29	4451	20.40	2567	11.77	1822	8.35	2256	10.34	1868	8.56	553	2.53	296	1.36	1358	6.22	1127	5.17	12252	56.16	9564	43.84	21816
2003	5462	24.99	4585	20.98	2587	11.83	1738	7.95	2237	10.23	1836	8.40	634	2.90	311	1.42	1329	6.08	1140	5.22	12249	56.04	9610	43.96	21859
2004	5643	25.81	4331	19.81	2618	11.97	1835	8.39	2144	9.80	1806	8.26	609	2.79	305	1.39	1410	6.45	1166	5.33	12424	56.82	9443	43.18	21867
2005	6099	26.08	4636	19.83	2620	11.20	1813	7.75	2388	10.21	1926	8.24	723	3.09	362	1.55	1577	6.74	1240	5.30	13407	57.33	9977	42.67	23384
2006	6146	25.81	4623	19.42	2828	11.88	1787	7.51	2286	9.60	1802	7.57	899	3.78	486	2.04	1745	7.33	1207	5.07	13904	58.40	9905	41.60	23809
2007	6380	25.85	4610	18.68	2917	11.82	1829	7.41	2261	9.16	1821	7.38	1069	4.33	561	2.27	1909	7.73	1326	5.37	14536	58.89	10147	41.11	24683
2008	6065	26.46	4344	18.95	2770	12.09	1743	7.61	2038	8.89	1636	7.14	979	4.27	508	2.22	1632	7.12	1203	5.25	13484	58.84	9434	41.16	22918
2009	5992	27.45	4124	18.89	2834	12.98	1753	8.03	1846	8.46	1437	6.58	805	3.69	457	2.09	1502	6.88	1078	4.94	12979	59.46	8849	40.54	21828
2010	5973	27.73	4157	19.30	2866	13.31	1706	7.92	1712	7.95	1413	6.56	842	3.91	437	2.03	1459	6.77	971	4.51	12852	59.68	8684	40.32	21536
2011	6025	28.77	3972	18.96	2792	13.33	1713	8.18	1605	7.66	1221	5.83	740	3.53	416	1.99	1495	7.14	965	4.61	12657	60.43	8287	39.57	20944
2012	6021	29.10	3863	18.67	2800	13.53	1668	8.06	1595	7.71	1195	5.78	754	3.64	406	1.96	1450	7.01	939	4.54	12620	60.99	8071	39.01	20691
2013	5974	28.74	3925	18.88	2711	13.04	1621	7.80	1628	7.83	1154	5.55	794	3.82	415	2.00	1569	7.55	998	4.80	12676	60.97	8113	39.03	20789
2014	5741	28.55	3709	18.44	2858	14.21	1666	8.28	1452	7.22	1115	5.54	730	3.63	401	1.99	1504	7.48	934	4.64	12285	61.09	7825	38.91	20110
2015	5953	29.00	3799	18.51	2907	14.16	1733	8.44	1424	6.94	971	4.73	743	3.62	377	1.84	1629	7.94	993	4.84	12656	61.65	7873	38.35	20529
2016	5954	29.29	3763	18.51	2907	14.30	1618	7.96	1380	6.79	938	4.61	814	4.00	409	2.01	1633	8.03	912	4.49	12688	62.42	7640	37.58	20328
2017	5736	29.74	3547	18.39	2726	14.13	1663	8.62	1174	6.09	861	4.46	695	3.60	342	1.77	1629	8.45	913	4.73	11960	62.01	7326	37.99	19286
2018	5599	29.43	3391	17.82	2843	14.94	1581	8.31	1078	5.67	793	4.17	646	3.40	374	1.97	1780	9.36	939	4.94	11946	62.79	7078	37.21	19024

Table S3. Number of deaths among women in urban and rural areas during 1990-2018.

Years	Cardiovascular diseases				Cancer				External causes				Gastrointestinal diseases				Others				All causes				All
	Urban		Rural		Urban		Rural		Urban		Rural		Urban		Rural		Urban		Rural		Urban		Rural		
	N	%	N	%	N	%	N	%	N	%	N	%	N	%	N	%	N	%	N	%	N	%	N	%	
1990	6365	33.25	6477	33.84	1926	10.06	1089	5.69	585	3.06	434	2.27	285	1.49	140	0.73	1011	5.28	829	4.33	10172	53.14	8969	46.86	19141
1991	6314	32.74	6262	32.47	1978	10.26	1121	5.81	644	3.34	450	2.33	251	1.30	140	0.73	1170	6.07	957	4.96	10357	53.70	8930	46.30	19287
1992	6208	32.18	5945	30.82	1900	9.85	1201	6.23	664	3.44	478	2.48	288	1.49	159	0.82	1247	6.46	1201	6.23	10307	53.43	8984	46.57	19291
1993	6794	31.87	6768	31.75	2030	9.52	1257	5.90	827	3.88	483	2.27	256	1.20	161	0.76	1300	6.10	1439	6.75	11207	52.58	10108	47.42	21315
1994	6907	32.65	6256	29.58	2083	9.85	1166	5.51	902	4.26	529	2.50	306	1.45	150	0.71	1367	6.46	1486	7.03	11565	54.68	9587	45.32	21152
1995	7008	33.33	6655	31.66	2073	9.86	1167	5.55	840	4.00	534	2.54	331	1.57	169	0.80	1278	6.08	968	4.60	11530	54.84	9493	45.16	21023
1996	6674	33.29	6433	32.09	2116	10.55	1149	5.73	797	3.98	545	2.72	290	1.45	178	0.89	1057	5.27	809	4.04	10934	54.54	9114	45.46	20048
1997	6607	33.81	6399	32.75	2072	10.60	1124	5.75	695	3.56	480	2.46	317	1.62	178	0.91	937	4.80	732	3.75	10628	54.39	8913	45.61	19541
1998	6591	33.69	6279	32.09	2128	10.88	1199	6.13	720	3.68	447	2.28	379	1.94	204	1.04	924	4.72	693	3.54	10742	54.91	8822	45.09	19564
1999	6429	33.78	5922	31.12	2160	11.35	1229	6.46	698	3.67	479	2.52	397	2.09	167	0.88	867	4.56	683	3.59	10551	55.44	8480	44.56	19031
2000	6266	33.85	5588	30.19	2169	11.72	1234	6.67	672	3.63	485	2.62	378	2.04	192	1.04	882	4.76	645	3.48	10367	56.00	8144	44.00	18511
2001	6475	34.39	5716	30.36	2277	12.09	1155	6.13	716	3.80	452	2.40	417	2.21	200	1.06	850	4.51	570	3.03	10735	57.02	8093	42.98	18828
2002	6630	34.43	5732	29.77	2242	11.64	1239	6.43	688	3.57	467	2.43	438	2.27	223	1.16	925	4.80	672	3.49	10923	56.73	8333	43.27	19256
2003	6473	33.84	5775	30.19	2300	12.02	1214	6.35	682	3.56	486	2.54	452	2.36	212	1.11	926	4.84	611	3.19	10833	56.63	8298	43.37	19131
2004	6780	34.82	5777	29.67	2297	11.80	1209	6.21	655	3.36	468	2.40	458	2.35	260	1.34	936	4.81	633	3.25	11126	57.14	8347	42.86	19473
2005	7073	34.65	6015	29.46	2387	11.69	1228	6.02	701	3.43	534	2.62	502	2.46	298	1.46	997	4.88	680	3.33	11660	57.11	8755	42.89	20415
2006	7533	35.86	6019	28.66	2332	11.10	1201	5.72	735	3.50	513	2.44	615	2.93	341	1.62	1075	5.12	640	3.05	12290	58.51	8714	41.49	21004
2007	7398	35.33	5919	28.27	2355	11.25	1181	5.64	654	3.12	510	2.44	709	3.39	386	1.84	1144	5.46	685	3.27	12260	58.55	8681	41.45	20941
2008	7358	35.18	5856	28.00	2545	12.17	1208	5.78	649	3.10	462	2.21	680	3.25	377	1.80	1147	5.48	632	3.02	12379	59.19	8535	40.81	20914
2009	7464	36.94	5712	28.27	2406	11.91	1137	5.63	500	2.47	385	1.91	608	3.01	353	1.75	1015	5.02	624	3.09	11993	59.36	8211	40.64	20204
2010	7660	37.21	5837	28.36	2342	11.38	1196	5.81	554	2.69	366	1.78	609	2.96	329	1.60	1097	5.33	594	2.89	12262	59.57	8322	40.43	20584
2011	7537	37.51	5552	27.63	2450	12.19	1151	5.73	548	2.73	346	1.72	608	3.03	294	1.46	1022	5.09	585	2.91	12165	60.54	7928	39.46	20093
2012	7884	38.94	5402	26.68	2365	11.68	1165	5.75	526	2.60	344	1.70	559	2.76	346	1.71	1076	5.31	580	2.86	12410	61.29	7837	38.71	20247
2013	7921	38.23	5541	26.74	2415	11.65	1125	5.43	499	2.41	327	1.58	682	3.29	343	1.66	1210	5.84	659	3.18	12727	61.42	7995	38.58	20722
2014	7831	38.88	5241	26.02	2412	11.97	1091	5.42	479	2.38	288	1.43	629	3.12	330	1.64	1187	5.89	654	3.25	12538	62.25	7604	37.75	20142
2015	8254	38.85	5582	26.27	2538	11.95	1170	5.51	506	2.38	298	1.40	636	2.99	342	1.61	1260	5.93	661	3.11	13194	62.10	8053	37.90	21247
2016	8194	39.44	5192	24.99	2543	12.24	1129	5.43	481	2.31	263	1.27	620	2.98	322	1.55	1356	6.53	678	3.26	13194	63.50	7584	36.50	20778
2017	8049	38.59	5176	24.82	2440	11.70	1167	5.60	509	2.44	264	1.27	614	2.94	342	1.64	1544	7.40	751	3.60	13156	63.08	7700	36.92	20856
2018	7975	38.81	4953	24.10	2487	12.10	1116	5.43	478	2.33	260	1.27	591	2.88	289	1.41	1614	7.85	787	3.83	13145	63.97	7405	36.03	20550

Table S4. Age-standardized mortality rates (ASMR) from all causes and major causes among men in 1990-2018 (100,000 population).

Years	Cardiovascular diseases		Cancer		External causes		Gastrointestinal diseases		All causes	
	Urban ASMR (95% CI)	Rural ASMR (95% CI)	Urban ASMR (95% CI)	Rural ASMR (95% CI)	Urban ASMR (95% CI)	Rural ASMR (95% CI)	Urban ASMR (95% CI)	Rural ASMR (95% CI)	Urban ASMR (95% CI)	Rural ASMR (95% CI)
1990	720.32 (700.08; 740.56)	758.33 (736.63; 780.03)	288.4 (275.84; 300.96)	283.59 (269.45; 297.73)	178.67 (170.15; 187.19)	276.01* (261.55; 290.47)	32.49 (28.39; 36.59)	27.27 (22.81; 31.73)	1367.07 (1340.49; 1393.65)	1567.25* (1535.25; 1599.25)
1991	691.84 (672.34; 711.34)	771.10* (749.2; 793.00)	287.79 (275.53; 300.05)	277.25 (263.37; 291.13)	205.54 (196.6; 214.48)	333.81* (317.87; 349.75)	39.63 (35.15; 44.11)	31.86 (27.02; 36.7)	1395.85 (1369.59; 1422.11)	1650.85* (1617.95; 1683.75)
1992	696.6 (677.28; 715.92)	766.92* (745.02; 788.82)	287.46 (275.3; 299.62)	313 (298.26; 327.74)	210.44 (201.36; 219.52)	324.47* (308.73; 340.21)	44.46 (39.72; 49.2)	33.67 (28.69; 38.65)	1422.35 (1396.09; 1448.61)	1688.15* (1654.91; 1721.39)
1993	774.09 (754.01; 794.17)	871.33* (847.95; 894.71)	295.75 (283.37; 308.13)	305.39 (290.83; 319.95)	269.12 (258.84; 279.4)	367.88* (351.08; 384.68)	43.31 (38.69; 47.93)	40.31 (34.81; 45.81)	1580.76 (1553.4; 1608.12)	1879.65* (1844.57; 1914.73)
1994	776.05 (755.95; 796.15)	832.19* (809.25; 855.13)	297.17 (284.87; 309.47)	300.82 (286.34; 315.3)	294.96 (284.24; 305.68)	425.00* (407; 443)	48.86 (44.04; 53.68)	39.33 (33.89; 44.77)	1642.85 (1615.07; 1670.63)	1896* (1860.56; 1931.44)
1995	717.64 (698.34; 736.94)	834.57* (811.49; 857.65)	304.99 (292.57; 317.41)	306.46 (291.82; 321.1)	283.00 (272.42; 293.58)	405.56* (387.88; 423.24)	51.54 (46.64; 56.44)	41.36 (35.74; 46.98)	1574.75 (1547.61; 1601.89)	1850.1* (1814.86; 1885.34)
1996	696.49 (677.61; 715.37)	836.55* (813.39; 859.71)	289.4 (277.54; 301.26)	311.92 (297.02; 326.82)	238.51 (228.87; 248.15)	365.53* (348.81; 382.25)	45.8 (41.16; 50.44)	40.69 (35.17; 46.21)	1460.92 (1434.92; 1486.92)	1797.5* (1762.74; 1832.26)
1997	664.65 (646.35; 682.95)	772.20* (749.84; 794.56)	284.88 (273.18; 296.58)	307.37 (292.57; 322.17)	228.27 (218.75; 237.79)	351.5* (335.04; 367.96)	47.05 (42.39; 51.71)	40.69 (35.19; 46.19)	1391.18 (1365.92; 1416.44)	1694.92* (1661.04; 1728.8)
1998	636.53 (618.73; 654.33)	745.50* (723.52; 767.48)	284.68 (273.08; 296.28)	312.07* (297.21; 326.93)	221.37 (211.95; 230.79)	350.58* (334.26; 366.9)	50.39 (45.67; 55.11)	47.94 (41.94; 53.94)	1345.14 (1320.4; 1369.88)	1678.31* (1644.69; 1711.93)
1999	641.88 (624.12; 659.64)	759.53* (737.25; 781.81)	284.22 (272.72; 295.72)	322.15* (307.11; 337.19)	207.95 (198.85; 217.05)	357.50* (341.02; 373.98)	48.50 (43.84; 53.16)	41.6 (35.98; 47.22)	1316.74 (1292.38; 1341.1)	1676.4* (1642.82; 1709.98)
2000	615.80 (598.48; 633.12)	706.55* (684.97; 728.13)	290.25 (278.67; 301.83)	302.81 (288.25; 317.37)	215.33 (206.09; 224.57)	316.58* (301.12; 332.04)	51.76 (46.96; 56.56)	39.26* (33.86; 44.66)	1315.03 (1290.79; 1339.27)	1560.32* (1527.86; 1592.78)
2001	647.98 (630.36; 665.60)	743.57* (721.49; 765.65)	289.8 (278.34; 301.26)	298.38 (284.02; 312.74)	232.95 (223.31; 242.59)	355.31* (338.91; 371.71)	58.03 (52.97; 63.09)	49.42 (43.36; 55.48)	1369.68 (1345.12; 1394.24)	1646.72* (1613.46; 1679.98)
2002	661.56 (643.78; 679.34)	774.71* (752.05; 797.37)	280.6 (269.38; 291.82)	307.32* (292.76; 321.88)	218.16 (208.82; 227.5)	347.53* (331.29; 363.77)	57.38 (52.42; 62.34)	53.63 (47.31; 59.95)	1368.14 (1343.64; 1392.64)	1683.61* (1649.93; 1717.29)
2003	643.75 (626.25; 661.25)	803.73* (780.47; 826.99)	280.71 (269.49; 291.93)	294.47 (280.13; 308.81)	216.39 (207.11; 225.67)	339.83* (323.81; 355.85)	64.98 (59.74; 70.22)	55.53 (49.13; 61.93)	1351.43 (1327.17; 1375.69)	1694.73* (1660.85; 1728.61)
2004	645.43 (628.07; 662.79)	758.37* (735.49; 781.25)	278.06 (267; 289.12)	309.79 * (295.07; 324.51)	208.9 (199.76; 218.04)	333.39* (317.55; 349.23)	61.7 (56.6; 66.8)	54.55 (48.21; 60.89)	1346.02 (1321.94; 1370.1)	1664.38* (1630.56; 1698.2)
2005	682.3 (664.7; 699.9)	816.43* (792.61; 840.25)	272.95 (262.13; 283.77)	306.82* (292.14; 321.5)	234.43 (224.73; 244.13)	359.96* (343.4; 376.52)	73.45 (67.89; 79.01)	65.23 (58.27; 72.19)	1432.05 (1407.45; 1456.65)	1773.87* (1738.83; 1808.91)
2006	671.25 (654.01; 688.49)	809.26* (785.6; 832.92)	291.62 (280.5; 302.74)	304.54 (289.86; 319.22)	227.37 (217.77; 236.97)	336.24* (320.24; 352.24)	90.29 (84.17; 96.41)	86.14 (78.22; 94.06)	1466.32 (1441.62; 1491.02)	1754.22* (1719.4; 1789.04)
2007	682.36 (665.24; 699.48)	809.15* (785.41; 832.89)	297.62 (286.46; 308.78)	311.67 (296.83; 326.51)	224.05 (214.55; 233.55)	344.15* (327.85; 360.45)	106.06 (99.48; 112.64)	102.08 (93.36; 110.8)	1511.21 (1486.41; 1536.01)	1802.64* (1767.26; 1838.02)
2008	641.75 (625.29; 658.21)	759.74* (736.78; 782.7)	280.47 (269.67; 291.27)	300.1 (285.44; 314.76)	204.2 (195.08; 213.32)	313.38* (297.74; 329.02)	96.41 (90.17; 102.65)	92.16 (83.88; 100.44)	1394.17 (1370.45; 1417.89)	1687.2* (1652.86; 1721.54)
2009	621.46 (605.44; 637.48)	713.87* (691.73; 736.01)	285.7 (274.84; 296.56)	299.5 (284.94; 314.06)	184.31 (175.69; 192.93)	273.78* (259.2; 288.36)	80.7 (74.96; 86.44)	83.80 (75.84; 91.76)	1327.39 (1304.39; 1350.39)	1562.72* (1529.86; 1595.58)
2010	606.05 (590.39; 621.71)	726.64* (704.32; 748.96)	285.23 (274.45; 296.01)	292.8 (278.42; 307.18)	174.97 (166.47; 183.47)	272.01* (257.41; 286.61)	85.89 (79.91; 91.87)	81.36 (73.48; 89.24)	1301.39 (1278.75; 1324.03)	1548.16* 1515.4; 1580.92)
2011	596.45 (581.09; 611.81)	694.2* (672.34; 716.06)	271.32 (260.92; 281.72)	298.26* (283.68; 312.84)	166.14 (157.8; 174.48)	234.1* (220.56; 247.64)	75.22 (69.64; 80.8)	76.25 (68.69; 83.81)	1262.49 (1240.29; 1284.69)	1478.29* (1446.23; 1510.35)
2012	586.59 (571.53; 601.65)	668.92* (647.5; 690.34)	270.71 (260.35; 281.07)	287.12 (272.88; 301.36)	166.68 (158.3; 175.06)	232.59* (218.99; 246.19)	76.94 (71.28; 82.6)	75.56 (67.98; 83.14)	1247.13 (1225.19; 1269.07)	1433.13* (1401.55; 1464.71)
2013	570.13 (555.47; 584.79)	673.34* (652.08; 694.6)	260.01 (249.91; 270.11)	280.45 (266.35; 294.55)	170.42 (161.94; 178.9)	224.39* (211.01; 237.77)	80.40 (74.64; 86.16)	76.41 (68.81; 84.01)	1237.79 (1216.09; 1259.49)	1434.91* (1403.49; 1466.33)
2014	539.32 (525.2; 553.44)	631.45* (610.91; 651.99)	270.93 (260.71; 281.15)	287.05 (272.85; 301.25)	153.11 (145.03; 161.19)	217.42* (204.22; 230.62)	73.83 (68.31; 79.35)	72.09 (64.79; 79.39)	1184.51 (1163.43; 1205.59)	1377.47* (1346.67; 1408.27)
2015	548.57	646.03*	274.5	295.58	149.92	190.89*	75.06	68.58	1207.18	1377.35*

	(534.47; 562.67)	(625.31; 666.75)	(264.24; 284.76)	(281.28; 309.88)	(141.94; 157.9)	(178.43; 203.35)	(69.5; 80.62)	(61.42; 75.74)	(1186; 1228.36)	(1346.77; 1407.93)
2016	544.04 (530.06; 558.02)	630.6* (610.32; 650.88)	273.73 (263.49; 283.97)	276.63 (262.77; 290.49)	146.14 (138.2; 154.08)	182.94* (170.78; 195.1)	82.39 (76.57; 88.21)	72.76 (65.46; 80.06)	1207.04 (1185.88; 1228.2)	1327.3* (1297.34; 1357.26)
2017	515.10 (501.58; 528.62)	589.78* (570.24; 609.32)	253.64 (243.82; 263.46)	280.23* (266.39; 294.07)	124.30 (116.96; 131.64)	169.57* (157.79; 181.35)	70.20 (64.82; 75.58)	61.51 (54.71; 68.31)	1118.62 (1098.38; 1138.86)	1261.28* (1232.22; 1290.34)
2018	498.80 (485.54; 512.06)	558.66* (539.72; 577.6)	264.34 (254.32; 274.36)	263.84 (250.46; 277.22)	114.44 (107.38; 121.5)	152.03* (140.99; 163.07)	64.32 (59.2; 69.44)	68.68 (61.42; 75.94)	1112.12 (1091.94; 1132.3)	1205.8* (1177.48; 1234.12)

CI, confidence intervals.

*$p < 0.05$ compared to urban areas.

Table S5. Age-standardized mortality rates (ASMR) from all causes and major causes among women in 1990-2018 (100,000 population).

Years	Cardiovascular diseases		Cancer		External causes		Gastrointestinal diseases		All causes	
	Urban ASMR (95% CI)	Rural ASMR (95% CI)	Urban ASMR (95% CI)	Rural ASMR (95% CI)	Urban ASMR (95% CI)	Rural ASMR (95% CI)	Urban ASMR (95% CI)	Rural ASMR (95% CI)	Urban ASMR (95% CI)	Rural ASMR (95% CI)
1990	446.25 (435.15; 457.35)	528.95* (515.57; 542.33)	149.46 (142.54; 156.38)	126.41* (118.13; 134.69)	44.02 (40.34; 47.7)	64.22* (57.58; 70.86)	21.51 (18.91; 24.11)	15.11* (12.33; 17.89)	740.25 (725.67; 754.83)	828.75* (810.17; 847.33)
1991	471.54 (459.70; 483.38)	513.31* (500.03; 526.59)	151.52 (144.62; 158.42)	128.03* (119.79; 136.27)	48.35 (44.49; 52.21)	68.55* (61.61; 75.49)	18.66 (16.26; 21.06)	14.48 (11.76; 17.2)	743.14 (728.62; 757.66)	833.91* (815.07; 852.75)
1992	420.76 (410.16; 431.36)	481.45* (468.63; 494.27)	143.76 (137.1; 150.42)	136.16 (127.62; 144.7)	49.71 (45.81; 53.61)	72.87* (65.73; 80.01)	21.25 (18.71; 23.79)	14.84* (11.92; 17.76)	730.31 (716.01; 744.61)	830.5* (811.72; 849.28)
1993	458.01 (446.99; 469.03)	547.99* (534.39; 561.59)	153.2 (146.34; 160.06)	141.54 (132.88; 150.2)	61.53 (57.17; 65.89)	75.04* (67.76; 82.32)	19.20 (16.76; 21.64)	16.83 (13.91; 19.75)	789.34 (774.52; 804.16)	928.69* (908.99; 948.39)
1994	463.79 (452.71; 474.87)	520.27* (506.71; 533.83)	155.15 (148.27; 162.03)	131.59* (123.19; 139.99)	68.45 (63.83; 73.07)	83.55* (75.79; 91.31)	22.48 (19.88; 25.08)	16.92 (13.84; 20)	812.99 (797.95; 828.03)	909.47* (889.47; 929.47)
1995	459.74 (448.9; 470.58)	553.49* (539.65; 567.33)	152.13 (145.37; 158.89)	129.46* (121.16; 137.76)	63.97 (59.51; 68.43)	83.19* (75.45; 90.93)	23.81 (21.15; 26.47)	19.46 (16.16; 22.76)	799.36 (784.56; 814.16)	897.88* (878.12; 917.64)
1996	429.58 (419.24; 439.92)	535.95* (522.41; 549.49)	152 (145.32; 158.68)	131.37* (122.87; 139.87)	59.92 (55.62; 64.22)	86.06* (78.12; 94)	20.46 (18.02; 22.9)	19.26 (16.02; 22.5)	743.51 (729.39; 757.63)	870.87* (851.31; 890.43)
1997	413.87 (403.93; 423.81)	533.39* (520.03; 546.75)	145.62 (139.16; 152.08)	127.73* (119.33; 136.13)	52.09 (48.07; 56.11)	75.64* (68.22; 83.06)	21.84 (19.36; 24.32)	20.28 (16.86; 23.7)	705.09 (691.55; 718.63)	847.63* (828.49; 866.77)
1998	409.84 (399.98; 419.7)	528.13* (514.73; 541.53)	146.95 (140.51; 153.39)	136.18 (127.48; 144.88)	53.93 (49.83; 58.03)	71.22* (63.98; 78.46)	26.33 (23.59; 29.07)	22.11 (18.67; 25.55)	705.49 (692.01; 718.97)	840.81* (821.75; 859.87)
1999	391.68 (382.18; 401.18)	498.92* (485.78; 512.06)	147.11 (140.69; 153.53)	138.81 (130.09; 147.53)	51.48 (47.52; 55.44)	78.25* (70.61; 85.89)	27.12 (24.36; 29.88)	19.98* (16.52; 23.44)	680.65 (667.53; 693.77)	818.61* (799.53; 837.69)
2000	375.62 (366.38; 384.86)	467.27* (454.55; 479.99)	145.44 (139.1; 151.78)	138.52 (129.78; 147.26)	49.54 (45.66; 53.42)	76.53* (69.09; 83.97)	25.43 (22.77; 28.09)	20.66 (17.34; 23.98)	661.38 (648.48; 674.28)	779.34* (760.74; 797.94)
2001	380.99 (371.75; 390.23)	472.83* (460.05; 485.61)	149.11 (142.75; 155.47)	129.52* (121.02; 138.02)	53.43 (49.35; 57.51)	70.27* (63.11; 77.43)	28.28 (25.46; 31.1)	23.79 (20.01; 27.57)	671.97 (659.07; 684.87)	762.99* (744.61; 781.37)
2002	390.42 (381.06; 399.78)	481.41* (468.55; 494.27)	145.18 (138.9; 151.46)	135.95 (127.37; 144.53)	49.84 (45.94; 53.74)	72.49* (65.23; 79.75)	28.59 (25.79; 31.39)	25.42 (21.64; 29.2)	678.83 (665.89; 691.77)	794.75* (776.01; 813.49)
2003	367.46 (358.46; 376.46)	481.77* (468.75; 494.79)	145.16 (138.92; 151.4)	133.71 (125.07; 142.35)	48.90 (45.04; 52.76)	76.32* (68.84; 83.8)	29.38 (26.54; 32.22)	23.27 (19.65; 26.89)	654.17 (641.53; 666.81)	788.38* (769.54; 807.22)
2004	369.32 (360.42; 378.22)	482.7* (469.56; 495.84)	142.22 (136.06; 148.38)	134.23 (125.53; 142.93)	47.02 (43.2; 50.84)	73.64* (66.3; 80.98)	28.72 (25.96; 31.48)	28.51 (24.57; 32.45)	649.84 (637.32; 662.36)	794.87* (775.85; 813.89)
2005	371.85 (363.05; 380.65)	500.1* (486.72; 513.48)	143.69 (137.57; 149.81)	131.23 (122.75; 139.71)	49.83 (45.95; 53.71)	87.45 (79.33; 95.57)	31.74 (28.82; 34.66)	36.52 (31.82; 41.22)	663.48 (650.92; 676.04)	833.23* (813.69; 852.77)
2006	382.74 (373.9; 391.58)	493.89* (480.51; 507.27)	135.94 (130.04; 141.84)	131.44 (122.82; 140.06)	53.69 (49.55; 57.83)	82.10* (74.26; 89.94)	38.84 (35.6; 42.08)	43.12 (37.96; 48.28)	682.58 (669.86; 695.3)	827.46* (807.78; 847.14)
2007	361.74 (353.24; 370.24)	477.96* (464.74; 491.18)	137.33 (131.39; 143.27)	128.3 (119.8; 136.8)	47.33 (43.45; 51.21)	80.08* (72.4; 87.76)	45.38 (41.86; 48.9)	49.53 (43.97; 55.09)	668.31 (655.75; 680.87)	814.01* (794.51; 833.51)
2008	340.89 (332.85; 348.93)	464.44* (451.5; 477.38)	148.32 (142.12; 154.52)	133.55 (124.75; 142.35)	46.77 (42.95; 50.59)	73.99* (66.51; 81.47)	42.89 (39.47; 46.31)	48.07 (42.55; 53.59)	651.69 (639.45; 663.93)	793.99* (774.67; 813.31)
2009	329.82 (322.04; 337.6)	444.46* (431.9; 457.02)	137.39 (131.49; 143.29)	127.70 (119.1; 136.3)	35.53 (32.17; 38.89)	62.25* (55.35; 69.15)	36.00 (32.92; 39.08)	39.41 (34.61; 44.21)	599.62 (588.14; 611.1)	745.84* (727.3; 764.38)
2010	328.22 (320.56; 335.88)	438.68* (426.38; 450.98)	128.69 (123.03; 134.35)	133.46 (124.72; 142.2)	39.63 (36.07; 43.19)	56.38* (49.92; 62.84)	35.17 (32.13; 38.21)	37.33 (32.63; 42.03)	596.16 (584.78; 607.54)	729.2* (711.18; 747.22)
2011	310.44 (303.08; 317.8)	413.01* (401.03; 424.99)	135.91 (130.05; 141.77)	130.71 (121.97; 139.45)	37.98 (34.52; 41.44)	53.31* (46.95; 59.67)	34.57 (31.55; 37.59)	34.80 (30.18; 39.42)	575.46 (564.36; 586.56)	701.43* (683.37; 719.49)
2012	313.19 (305.91; 320.47)	391.51* (379.99; 403.03)	129.69 (123.95; 135.43)	124.78 (116.46; 133.1)	35.89 (32.53; 39.25)	55.82* (49.24; 62.4)	31.39 (28.51; 34.27)	39.45* (34.57; 44.33)	569.668 (558.688; 580.648)	673.58* (656.16; 691)
2013	304.42 (297.3; 311.54)	393.69* (382.15; 405.23)	129.56 (123.86; 135.26)	121.81 (113.55; 130.07)	34.4 (31.08; 37.72)	52.28* (45.9; 58.66)	38.20 (35; 41.4)	37.42 (32.72; 42.12)	571.44 (560.46; 582.42)	674.63* (657.23; 692.03)
2014	293.48 (286.54; 300.42)	367.3* (356.28; 378.32)	126.32 (120.74; 131.9)	122.05 (113.65; 130.45)	32.39 (29.17; 35.61)	44.84* (38.84; 50.84)	33.68 (30.72; 36.64)	36.45 (31.77; 41.13)	548.02 (537.34; 558.7)	641.54* (624.44; 658.64)
2015	297.54	391.28*	132.57	131.19	33.25	45.72*	34.6	36.57	562.01	673.77*

	(290.7; 304.38)	(379.92; 402.64)	(126.83; 138.31)	(122.47; 139.91)	(29.97; 36.53)	(39.72; 51.72)	(31.56; 37.64)	(31.95; 41.19)	(551.27; 572.75)	(656.41; 691.13)
2016	289.88<br>(283.12; 296.64)	365.02*<br>(354; 376.04)	133.74<br>(127.92; 139.56)	123.35<br>(115.03; 131.67)	30.77<br>(27.63; 33.91)	38<br>(32.64; 43.36)	33.41<br>(30.41; 36.41)	34.38<br>(29.88; 38.88)	554.89<br>(544.17; 565.61)	631.8*<br>(615.08; 648.52)
2017	273.34<br>(266.92; 279.76)	360.4*<br>(349.48; 371.32)	124.54<br>(118.96; 130.12)	131.34<br>(122.54; 140.14)	32.21<br>(28.97; 35.45)	37.04<br>(31.76; 42.32)	31.18<br>(28.3; 34.06)	35.13<br>(30.65; 39.61)	534.49<br>(524.09; 544.89)	633.95*<br>(617.25; 650.65)
2018	267.77<br>(261.35; 274.19)	338.8*<br>(328.42; 349.18)	125.92<br>(120.32; 131.52)	123.05<br>(114.51; 131.59)	28.92<br>(25.88; 31.96)	36.9<br>(31.56; 42.24)	30.19<br>(27.37; 33.01)	29.09<br>(25.09; 33.09)	526.06<br>(515.7; 536.42)	607.97*<br>(591.51; 624.43)

CI, confidence intervals.

*$p < 0.05$ compared to urban areas.

Table S6. Trends in age-standardized mortality rates among men by place of residence during 1990-2018.

Causes of deaths	Area	Number of cut-off points	Years of cut-off point	Period 1		Period 2		Period 3		Period 4		All period (1990-2018)
				Years	AAC (95% CI), p	Years	AAC (95% CI), p	Years	AAC (95% CI), p	Years	AAC (95% CI), p	AAC (95% CI), p
Cardiovascular diseases	Urban	3	1994 1998 2006	1990-1994	2.48 (-0.16, 5.20), 0.06465	1994-1998	-4.87 (-8.72, -0.85), 0.02042	1998-2006	0.91 (-0.20, 2.04), 0.10167	2006-2018	-2.41 (-2.88, -1.93), 0.00000	-1.16 (-1.40, -0.91), 0.00000
	Rural	3	1994 2000 2005	1990-1994	3.39 (0.26; 6.60), 0.03465	1994-2000	-2.81 (-4.89; -0,68), 0.01245	2000-2005	2.68 (-0.42; 5.87), 0.08761	2005-2018	-2.69 (-3.19; -2.19), 0.00000	-1.00 (-1.32; -0.69), 0.00000
Cancer	Urban	0	-	-	-	-	-	-	-	-	-	-0.37 (-0.50; -0.24), 0.00000
	Rural	3	1992 2007 2015	1990-1992	5.45 (-1.50; 12.89), 0.12050	1992-2007	-0.06 (-0.38; 0.26), 0.11709	2007-2015	-0.83 (-1.73; 0.08), 0.10900	2015-2018	-2.10 (-5.38; 1.29), 0.45595	-0.27 (-0.46; -0.08), 0.00611
External causes	Urban	3	1994 1998 2006	1990-1994	13.63 (7.52; 20.08), 0.00009	1994-1998	-7.72 (-15.43; 0.70), 0.06950	1998-2006	0.70 (-1.62; 3.08), 0.54119	2006-2018	-4.95 (-5.92; -3.96), 0.00000	-2.05 (-2.69; -1.40), 0.00000
	Rural	3	1994 1997 2007	1990-1994	10.38 (5.58, 15.39), 0.00015	1994-1997	-5.66 (-18.02, 8.57), 0.39827	1997-2007	-0.49 (-1.75, 0.80), 0.43901	2007-2018	-6.69 (-7.57, -5.80), 0.00000	-2.56 (-3.29, -1.84), 0.00000
Gastrointestinal diseases	Urban	3	1992 2002 2007	1990-1992	15.90 (-7.76, 45.65), 0.19337	1992-2002	2.20 (0.09, 4.36), 0.04128	2002-2007	11.39 (3.63, 19.74), 0.00534	2007-2018	-3.04 (-4.52,-1.54), 0.00043	2.85 (2.05, 3.66), 0.00000
	Rural	3	1993 2004 2007	1990-1993	10.71 (-0.84, 23.60), 0.06841	1993-2004	3.30 (1.55, 5.09), 0.00075	2004-2007	20.69 (-3.17, 50.43), 0.09033	2007-2018	-3.65 (-5.07, -2.21), 0.00004	3.55 (2.63, 4.47), 0.00000
All causes	Urban	3	1994 1999 2006	1990-1994	4.91 (2.05, 7.85), 0.00164	1994-1999	-4.45 (-7.06, -1.78), 0.00252	1999-2006	1.63 (0.14, 3.14), 0.03303	2006-2018	-2.21 (-2.71, -1.70), 0.00000	-0.86 (-1.14, -0.57), 0.00000
	Rural	3	1994 2000 2006	1990-1994	4.99 (2.46, 7.60), 0.00045	1994-2000	-2.93 (-4.59, -1.23), 0.00179	2000-2006	1.81 (0.06, 3.58), 0.04278	2006-2018	-3.08 (-3.53, -2.63), 0.00000	-1.07 (-1.42, -0.72), 0.00000

AAC, average annual changes; CI, confidence intervals.

Table S7. Trends in age-standardized mortality rates among women by place of residence during 1990-2018.

**Causes of deaths**	**Area**	**Number of cut-off points**	**Years of cut-off point**	**Period 1**		**Period 2**		**Period 3**		**Period 4**		**All period (1990-2018)**
				**Years**	**ACC (95% CI), p**	**Years**	**ACC (95% CI), p**	**Years**	**ACC (95% CI), p**	**Years**	**ACC (95% CI), p**	**ACC (95% CI), p**
Cardiovascular diseases	Urban	3	1995<br>1999<br>2006	1990-1995	0.96 (-0.75; 2.70), 0.25625	1995-1999	-4.03 (-7.63; -0.29), 0.03602	1999-2006	-0.79 (-2.06; 0.50), 0.21654	2006-2018	-2.57 (-3.01; -2.13), 0.00000	-1.85 (-2.03; -1.67), 0.0000
	Rural	3	1997<br>2000<br>2005	1990-1997	0.91 (-0.47, 2.30), 0.18604	1997-2000	-4.86 (-14.16, 5.45), 0.25179	2000-2005	1.25 (-1.99, 4.60), 0.24373	2005-2018	-2.86 (-3.38, -2.33), 0.01626	-1.48 (-1.76, -1.21), 0.0000
Cancer	Urban	0	-	-	-	-	-	-	-	-	-	-0.69 (-0.83, -0.55), 0.00000
	Rural	0	-	-	-	-	-	-	-	-	-	-0.22 (-0.38, -0.06), 0.00911
External causes	Urban	3	1994<br>1997<br>2006	1990-1994	12.42 (6.24, 18.96), 0.00031	1994-1997	-7.83 (-22.93, 10.23), 0.35418	1997-2006	-1.19 (-3.10, 0.76), 0.21764	2006-2018	-4.25 (-5.26, -3.24), 0.00000	-2.30 (-2.87, -1.72), 0.00000
	Rural	3	1994<br>2002<br>2005	1990-1994	6.97 (1.28, 12.98), 0.01815	1994-2002	-2.06 (-4.29, 0.23), 0.07561	2002-2005	6.69 (-10.25, 26.83), 0.44473	2005-2018	-6.62 (-7.46, -5.77), 0.00000	-2.30 (-3.07, -1.53), 0.00000
Gastrointestinal diseases	Urban	3	2004<br>2007<br>2010	1990-2004	3.03 (1.91, 4.17), 0.00001	2004-2007	14.94 (-9.04, 45.23), 0.22953	2007-2010	-7.60 (-26.87, 16.75), 0.48991	2010-2018	-1.31 (-3.80, 1.24), 0.29430	2.27 (1.59, 2.96), 0.00000
	Rural	3	2003<br>2007<br>2010	1990-2003	3.92 (2.88, 4.96), 0.00000	2003-2007	19.57 (8.69, 31.54), 0.00084	2007-2010	-8.99 (-24.80, 10.15), 0.31660	2010-2018	-1.90 (-3.92, 0.16), 0.06887	3.74 (2.79, 4.69), 0.00000
All causes	Urban	3	1994<br>1999<br>2006	1990-1994	2.71 (0.04, 5.45), 0.04661	1994-1999	-3.57 (-6.07, -0.99), 0.00917	1999-2006	-0.28 (-1.67, 1.14), 0.68613	2006-2018	-1.97 (-2.45, -1.48), 0.00000	-1.38 (-1.57, -1.20), 0.00000
	Rural	3	1994<br>2001<br>2005	1990-1994	3.04 (0.45, 5.78), 0.02356	1994-2001	-2.57 (-3.90, -1.21), 0.00079	2001-2005	2.03 (-2.06, 6.28), 0.31894	2005-2018	-2.43 (-2.85, -2.02), 0.00000	-1.23 (-1.47, -0.98), 0.00000

AAC, average annual changes; CI, confidence interval.

Table S8. Age-standardized mortality rate differences (rural-urban) among men and women during 1990-2018 (100,000 population).

Years	Cardiovascular diseases		Cancer		External causes		Gastrointestinal diseases		All causes	
	Men	Women	Men	Women	Men	Women	Men	Women	Men	Women
1990	38.01<br>(36.55; 39.47)	82.7<br>(80,42; 84,98)	-4.81<br>(-6.39; -3.23)	-23,05<br>(-24,41; -21,69)	97.34<br>(91,4; 103,28)	20,2<br>(17,24; 23,16)	-5.22<br>(-5,58; -4,86)	-6,40<br>(-6,58; -6,22)	200,18<br>(194,76; 205,60)	88,5<br>(84,50; 92,5)
1991	79.26<br>(76.86; 81.66)	78.51<br>(40,33; 43,21)	-10.54<br>(-12.16; -8.92)	-23,49<br>(-24,83; -22,15)	128.27<br>(121,27; 135,27)	20,2<br>(17,12; 23,28)	-7.77<br>(-8,13; -7,41)	-4,18<br>(-4,5; -3,86)	255<br>(248,36; 261,64)	90,77<br>(86,45; 95,09)
1992	70.32<br>(67.74; 72.9)	60.69<br>(58,47; 62,91)	25.54<br>(22.96; 28.12)	-7,6<br>(-9,48; -5,72)	114.03<br>(107,37; 120,69)	23,16<br>(19,92; 26,4)	-10.79<br>(-11,03; -10,55)	-4,41<br>(-6,79; -6,03)	265,8<br>(258,82; 272,78)	100,19<br>(95,71; 104,67)
1993	97.24<br>(93.94; 100.54)	89.98<br>(87,4; 92,56)	9.64<br>(7.46; 11.82)	-11,66<br>(-13,46; -9,86)	98.76<br>(92,24; 105,28)	13,51<br>(10,59; 16,43)	-3<br>(-3,88; -2,12)	-2,37<br>(-2,85; -1,89)	298,89<br>(291,17; 306,61)	139,35<br>(134,47; 144,23)
1994	56.13<br>(53.30; 58.98)	56.48<br>(54; 58,96)	3.65<br>(1.47; 5.83)	-23,56<br>(-25,08; -22,04)	130.04<br>(122,76; 137,32)	15,1<br>(11,96; 18,24)	-9.53<br>(-10,15; -8,91)	-5,56<br>(-6,04; -5,08)	253,15<br>(245,49; 260,81)	96,48<br>91,52; 101,44
1995	116.93<br>113.15; 120.71	93.75<br>(90,75; 96,75)	1.47<br>(-0.75; 3.69)	-22,67<br>(-24,21; -21,13)	122.56<br>(115,46; 129,66)	19,22<br>(15,94; 22,5)	-10.18<br>(-10,90; -9,46)	-4,35<br>(-4,99; -3,71)	275,35<br>(267,25; 283,45)	98,52<br>(93,56; 103,48)
1996	140.06<br>(135.78; 144.34)	106.37<br>(103,17; 109,57)	22.52<br>(19.48; 25.56)	-20,63<br>(-22,45; -18,81)	127.02<br>(119,94; 134,10)	26,14<br>(22,5; 29,78)	-5.11<br>(-5,99; -4,23)	-1,20<br>(-2; -0,4)	336,58<br>(327,82; 345,34)	127,36<br>(121,92; 132,8)
1997	107.55<br>(103.49; 111.61)	119.52<br>(116,1; 122,94)	22.49<br>(19.39; 25.59)	-17,89<br>(-19,83; -15,95)	123.23<br>(116,29; 130,17)	23,55<br>(20,15; 26,95)	-6.36<br>(-7,20; -5,52)	-1,56<br>(-2,5; -0,62)	303,74<br>(295,12; 312,36)	142,54<br>(136,94; 148,14)
1998	108.97<br>(104.79; 113.15)	118.29<br>(114,75; 121,83)	27.39<br>(24.13; 30.65)	-10,77<br>(-13,03; -8,51)	129.21<br>(122,31; 136,11)	17,29<br>(14,15; 20,43)	-2.45<br>(-3,73; -1,17)	-4,22<br>(-4,92; -3,52)	333,17<br>(324,29; 342,05)	135,32<br>(129,74; 140,9)
1999	117.65<br>(113.13; 122.17)	107.24<br>(103,6; 110,88)	37.93<br>(34.39; 41.47)	-8,3<br>(-10,6; -6)	149.55<br>(142,17; 156,93)	26,77<br>(23,09; 30,45)	-6.9<br>(-7,86; -5,94)	-7,14<br>-7,84; -6,44	359,66<br>(350,44; 368,88)	137,96<br>(132; 143,92)
2000	90.75<br>(86.49; 95.01)	91.65<br>(88,17; 95,13)	12.56<br>(9.58; 15.54)	-6,92<br>(-9,32; -4,52)	101.25<br>(95,03; 107,47)	27,02<br>(23,43; 30,55)	-12.5<br>(-13,10; -11,90)	-4,77<br>(-5,43; -4,11)	245,29<br>(237,07; 253,51)	117,96<br>(112,26; 123,66)
2001	95.59<br>(91.13; 100.05)	91.84<br>(88,3; 95,38)	8.58<br>(5.68; 11.48)	-19,59<br>(-21,73; -17,45)	122.36<br>(115,6; 129,12)	16,84<br>(13,76; 19,92)	-8.61<br>(-9,61; -7,61)	-4,49<br>(-5,36; -3,53)	277,04<br>(268,34; 285,74)	91,02<br>(85,54; 96,5)
2002	113.15<br>(108.27; 118.03)	90.99<br>(87,49; 94,49)	26.72<br>(23.38; 30.06)	-9,23<br>(-11,53; -6,93)	129.37<br>(122,47; 136,27)	22,65<br>(19,29; 26,01)	-3.75<br>(-5,11; -2,39)	-3,17<br>(-4,15; -2,19)	315,47<br>(306,29; 324,65)	115,92<br>(110,12; 121,72)
2003	159.98<br>(154.22; 165.74)	114.31<br>(110,29; 118,33)	13.76<br>(10.64; 16.88)	-11,45<br>(-13,85; -9,05)	123.44<br>(116,7; 130,18)	27,42<br>(23,8; 31,04)	-9.45<br>(-10,61; -8,29)	-6,11<br>(-6,89; -5,33)	343,3<br>(333,68; 352,92)	134,21<br>(128,01; 140,41)
2004	112.94<br>(107.42; 118.46)	113.38<br>(109,14; 117,62)	31.73<br>(28.07; 35.39)	-7,99<br>(-10,53; -5,45)	124.49<br>(117,79; 131,19)	26,62<br>(23,1; 30,14)	-7.15<br>(-8,39; -5,91)	-0,21<br>(-1,39; 0,97)	318,36<br>(308,62; 328,1)	145,03<br>(138,53; 151,53)
2005	134.13<br>(127.91; 740.35)	128.25<br>(123,67; 132,83)	33.87<br>(30.01; 37.73)	-12,46<br>(-14,82; -10,1)	125.53<br>(118,67; 132,39)	37,62<br>(33,38; 41,86)	-8.22<br>(-9,62; -6,82)	4,78<br>(3; 6,56)	341,82<br>(331,38; 352,26)	169,75<br>(162,77; 176,73)
2006	138.01<br>(131.59; 144.43)	111.15<br>(106,61; 115,69)	12.92<br>(9.36; 16.48)	-4,5<br>(-7,22; -1,78)	108.87<br>(102,47; 115,27)	28,41<br>(24,71; 32,11)	-4.15<br>(-5,95; -2,35)	4,32<br>(2,36; 6,2)	287,9<br>(277,78; 298,02)	144,88<br>(137,92; 151,84)
2007	126.79<br>(120.17; 133.41)	116.22<br>(111,5; 120,94)	14.05<br>(10.37; 17.73)	-9,03<br>(-11,59; -6,47)	120.1<br>(113,3; 126,9)	32,75<br>(28,95; 36,55)	-3.98<br>(-6,12; -1,84)	4,15<br>(2,11; 6,19)	291,43<br>(280,85; 302,01)	145,7<br>(138,76; 152,64)
2008	117.99<br>(111.49; 124.49)	123.55<br>(118,65; 128,45)	19.63<br>(15.77; 23.49)	-14,77<br>(-17,37; -12,17)	109.18<br>(102,66; 115,7)	27,22<br>(23,56; 30,88)	-4.25<br>(-6,29; -2,21)	5,18<br>(3,08; 7,28)	293,03<br>(282,41; 303,65)	142,3<br>(135,22; 149,38)
2009	92.41<br>(86.29; 98.53)	114.64<br>(109,86; 119,42)	13.8<br>(10.10; 17.50)	-9,69<br>(-12,39; -6,99)	89.47<br>(83,51; 195,43)	26,72<br>(23,18; 30,26)	3.1<br>(0,88; 5,32)	3,41<br>(1,69; 5,13)	235,33<br>(225,47; 245,19)	146,22<br>(139,16; 153,28)
2010	120.59<br>(113.93; 127.25)	110.46<br>(105,82; 115,10)	7.57<br>(3.97; 10.62)	4,77<br>(1,69; 7,85)	97.04<br>(90,94; 103,14)	16,75<br>(13,85; 19,65)	-4.53<br>(-6,43; -2,63)	2,16<br>(0,5; 3,82)	246,77<br>(236,65; 256,89)	133,04<br>(126,4; 139,68)
2011	97.75<br>(91.25; 104.25)	102.57<br>(97,95; 107,19)	26.94<br>(22.76; 31.12)	-5,2<br>(-8,08; -2,32)	67.96<br>(62,76; 73,16)	15,33<br>(12,43; 18,23)	1.03<br>(-0,95; 3,01)	0,23<br>(-1,37; 1,83)	215,8<br>(205,94; 225,66)	125,97<br>(119,01; 132,93)
2012	82.33	78.32	16.41	-4,91	65.91	19,93	-1.38	8,06	186	103,91

	(75.97; 88.69)	(74,08; 82,56)	(12.53; 20.29)	(-7,49; -2,33)	(60,69; 71,13)	(16,71; 23,15)	(-3,30; 0,54)	(6,06; 10,06)	(176,36; 195,64)	(97,48; 110,4)
2013	103.21<br>(96.61; 109.81)	89.27<br>(84,85; 93,69)	20.44<br>(16.44; 24.44)	-7,75<br>(-10,31; -5,19)	53.97<br>(49,07; 58,87)	17,88<br>(14,82; 20,94)	-3.99<br>(-5,83; -2,15)	-0,78<br>(-2,28; 0,72)	197,12<br>(187,40; 206,84)	103,19<br>(96,77; 109,61)
2014	92.13<br>(85.71; 598.55)	73.82<br>(69,74; 77,90)	16.12<br>(12.14; 20.10)	-4,27<br>(-7,09; -1,45)	64.31<br>(59,19; 69,43)	12,45<br>(9,67; 15,23)	-1.74<br>(-3,52; 0,04)	2,77<br>(1,05; 4,49)	192,96<br>(183,24; 202,68)	93,52<br>(87,1; 99,94)
2015	97.46<br>(90.84; 104.08)	93.74<br>(89,22; 98,26)	21.08<br>(17.04; 25.12)	-1,38<br>(-4,36; 1,6)	40.97<br>(36,49; 45,45)	12,47<br>(9,75; 15,19)	-6.48<br>(-8,08; -4,88)	1,97<br>(0,39; 3,55)	170,17<br>(160,77; 179,57)	111,76<br>(105,14; 118,38)
2016	86.56<br>(80.26; 92.86)	75.14<br>(70,88; 79,40)	2.90<br>(-0.72; 6.52)	-10,39<br>(-12,89; -7,89)	36.8<br>(32,58; 41,02)	7,23<br>(5,01; 9,45)	-9.63<br>(-11,11; -8,15)	0,97<br>(-0,53; 2,47)	120,26<br>(111,46; 129,06)	76,91<br>(70,91; 82,91)
2017	74.68<br>(68.66; 80.7)	87.06<br>(82,56; 91,56)	26.59<br>(22.57; 30.61)	6,8<br>(3,58; 10,02)	45.27<br>(40,83; 49,71)	4,83<br>(2,79; 6,87)	-8.69<br>(-10,11; -7,27)	3,95<br>(2,35; 5,55)	142,66<br>(133,84; 151,48)	99,46<br>(93,16; 105,76)
2018	59.86<br>(54.18; 65.54)	71.03<br>(67,07; 74,99)	-0.50<br>(-3.86; 2.86)	-2,87<br>(-5,81; 0,07)	37.59<br>(33,61; 41,57)	7,98<br>(5,68; 10,28)	4.36<br>(2,22; 6,50)	-1,10<br>(-2,28; 0,08)	93,68<br>(85,54; 101,82)	81,91<br>(75,81; 88,01)

Table S9. Age-standardized mortality rate ratio (rural/urban) among men and women during 1990-2018.

Years	Cardiovascular diseases		Cancer		External causes		Gastrointestinal diseases		All causes	
	Men	Women	Men	Women	Men	Women	Men	Women	Men	Women
1990	1.05 (0.98;1.13)	1.19 (1.09;1.29)	0.98 (0.87;1.10)	0.85 (0.70;1.00)	1.54 (1.37;1.74)	1.46 (1.12;1.86)	0.84 (0.55;1.21)	0.70 (0.39;1.15)	1.15 (1.09;1.2)	1.12 (1.04;1.2)
1991	1.11 (1.04;1.2)	1.18 (1.08;1.29)	0.96 (0.85;1.08)	0.84 (0.70;1.00)	1.62 (1.45;1.81)	1.42 (1.1;1.79)	0.8 (0.54;1.13)	0.78 (0.41;1.26)	1.18 (1.13;1.24)	1.12 (1.05;1.2)
1992	1.10 (1.02;1.18)	1.14 (1.04;1.25)	1.09 (0.97;1.22)	0.95 (0.79;1.12)	1.54 (1.38;1.72)	1.47 (1.14;1.84)	0.76 (0.52;1.06)	0.79 (0.45;1.25)	1.19 (1.13;1.24)	1.14 (1.06;1.22)
1993	1.13 (1.05;1.2)	1.20 (1.1;1.3)	1.03 (0.92;1.15)	0.92 (0.78;1.09)	1.37 (1.23;1.51)	1.22 (0.96;1.53)	0.93 (0.66;1.26)	0.88 (0.5;1.39)	1.19 (1.14;1.24)	1.18 (1.1;1.25)
1994	1.07 (1;1.15)	1.12 (1.03;1.22)	1.01 (0.9;1.13)	0.85 (0.71;1.01)	1.44 (1.31;1.58)	1.22 (0.97;1.51)	0.80 (0.57;1.09)	0.75 (0.42;1.18)	1.15 (1.1;1.21)	1.12 (1.05;1.19)
1995	1.16 (1.09;1.24)	1.2 (1.11;1.31)	1.00 (0.89;1.12)	0.85 (0.71;1.01)	1.43 (1.3;1.58)	1.30 (1.03;1.61)	0.80 (0.57;1.08)	0.82 (0.48;1.25)	1.17 (1.12;1.23)	1.12 (1.05;1.2)
1996	1.2 (1.12;1.29)	1.25 (1.14;1.36)	1.08 (0.96;1.2)	0.86 (0.72;1.02)	1.53 (1.38;1.7)	1.44 (1.15;1.77)	0.89 (0.63;1.2)	0.94 (0.56;1.45)	1.23 (1.17;1.29)	1.17 (1.09;1.25)
1997	1.16 (1.08;1.25)	1.29 (1.18;1.4)	1.08 (0.96;1.21)	0.88 (0.73;1.04)	1.54 (1.38;1.71)	1.45 (1.14;1.82)	0.86 (0.62;1.17)	0.93 (0.56;1.41)	1.22 (1.16;1.28)	1.2 (1.12;1.29)
1998	1.17 (1.09;1.26)	1.29 (1.18;1.4)	1.10 (0.98;1.22)	0.93 (0.78;1.09)	1.58 (1.42;1.76)	1.32 (1.03;1.66)	0.95 (0.69;1.25)	0.84 (0.52;1.27)	1.25 (1.19;1.31)	1.19 (1.11;1.28)
1999	1.18 (1.1;1.27)	1.27 (1.16;1.39)	1.13 (1.01;1.26)	0.94 (0.79;1.11)	1.72 (1.55;1.91)	1.52 (1.2;1.89)	0.86 (0.62;1.16)	0.74 (0.44;1.12)	1.27 (1.21;1.34)	1.2 (1.12;1.29)
2000	1.15 (1.06;1.24)	1.24 (1.13;1.36)	1.04 (0.93;1.17)	0.95 (0.8;1.12)	1.47 (1.31;1.64)	1.55 (1.22;1.93)	0.76 (0.54;1.03)	0.81 (0.5;1.24)	1.19 (1.13;1.25)	1.18 (1.1;1.26)
2001	1.15 (1.07;1.23)	1.24 (1.13;1.36)	1.03 (0.91;1.15)	0.87 (0.73;1.03)	1.53 (1.37;1.69)	1.32 (1.02;1.66)	0.85 (0.62;1.12)	0.84 (0.53;1.24)	1.2 (1.14;1.26)	1.14 (1.06;1.22)
2002	1.17 (1.09;1.26)	1.23 (1.12;1.35)	1.10 (0.98;1.22)	0.94 (0.78;1.1)	1.59 (1.43;1.77)	1.45 (1.13;1.82)	0.93 (0.7;1.22)	0.89 (0.57;1.29)	1.23 (1.17;1.29)	1.17 (1.09;1.26)
2003	1.25 (1.16;1.34)	1.31 (1.2;1.43)	1.05 (0.93;1.17)	0.92 (0.77;1.09)	1.57 (1.41;1.75)	1.56 (1.22;1.95)	0.85 (0.64;1.11)	0.79 (0.5;1.17)	1.25 (1.2;1.32)	1.21 (1.12;1.29)
2004	1.17 (1.09;1.26)	1.31 (1.19;1.43)	1.11 (0.99;1.24)	0.94 (0.79;1.12)	1.60 (1.43;1.77)	1.57 (1.23;1.96)	0.88 (0.66;1.15)	0.99 (0.66;1.43)	1.24 (1.18;1.3)	1.22 (1.14;1.31)
2005	1.2 (1.12;1.28)	1.34 (1.23;1.47)	1.12 (1;1.26)	0.91 (0.76;1.08)	1.54 (1.38;1.7)	1.75 (1.40;2.15)	0.89 (0.68;1.13)	1.15 (0.81;1.59)	1.24 (1.18;1.3)	1.26* (1.17;1.34)
2006	1.21 (1.12;1.29)	1.29 (1.18;1.41)	1.04 (0.93;1.17)	0.97 (0.81;1.14)	1.48 (1.32;1.64)	1.53 (1.21;1.90)	0.95 (0.76;1.18)	1.11 (0.8;1.49)	1.2 (1.14;1.25)	1.21 (1.13;1.30)
2007	1.19 (1.11;1.27)	1.32 (1.2;1.44)	1.05 (0.93;1.17)	0.93 (0.78;1.11)	1.54 (1.38;1.71)	1.69 (1.34;2.10)	0.96 (0.78;1.17)	1.09 (0.81;1.44)	1.19 (1.14;1.25)	1.22 (1.14;1.30)
2008	1.18 (1.1;1.27)	1.36 (1.24;1.49)	1.07 (0.95;1.2)	0.90 (0.75;1.07)	1.53 (1.37;1.71)	1.58 (1.23;1.97)	0.96 (0.77;1.17)	1.12 (0.83;1.48)	1.21 (1.15;1.27)	1.22 (1.14;1.31)
2009	1.15 (1.07;1.24)	1.35 (1.22;1.48)	1.05 (0.93;1.17)	0.93 (0.77;1.1)	1.49 (1.31;1.67)	1.75 (1.34;2.24)	1.04 (0.82;1.28)	1.09 (0.77;1.48)	1.18 (1.12;1.24)	1.24 (1.16;1.34)
2010	1.2	1.34	1.03	1.04	1.55	1.42	0.95	1.06	1.19	1.22

	(1.11;1.29)	(1.21;1.47)	(0.91;1.15)	(0.87;1.22)	(1.38;1.75)	(1.07;1.83)	(0.75;1.17)	(0.74;1.45)	(1.13;1.25)	(1.14;1.32)
2011	1.16 (1.08;1.25)	1.33 (1.21;1.47)	1.1 (0.98;1.23)	0.96 (0.80;1.14)	1.41 (1.23;1.6)	1.40 (1.05;1.83)	1.01 (0.8;1.26)	1.01 (0.69;1.39)	1.17 (1.11;1.23)	1.22 (1.13;1.31)
2012	1.14 (1.06;1.23)	1.25 (1.13;1.38)	1.06 (0.94;1.19)	0.96 (0.80;1.14)	1.40 (1.22;1.59)	1.56 (1.17;2.01)	0.98 (0.77;1.23)	1.26 (0.88;1.7)	1.15 (1.09;1.21)	1.18 (1.09;1.28)
2013	1.18 (1.09;1.27)	1.29 (1.17;1.43)	1.08 (0.95;1.21)	0.94 (0.78;1.12)	1.32 (1.15;1.5)	1.52 (1.13;1.98)	0.95 (0.74;1.18)	0.98 (0.68;1.34)	1.16 (1.1;1.22)	1.18 (1.09;1.27)
2014	1.17 (1.08;1.27)	1.25 (1.13;1.39)	1.06 (0.94;1.19)	0.97 (0.80;1.15)	1.42 (1.23;1.62)	1.38 (1.00;1.84)	0.98 (0.76;1.23)	1.08 (0.75;1.48)	1.16 (1.1;1.23)	1.17 (1.08;1.26)
2015	1.18 (1.09;1.27)	1.32 (1.19;1.45)	1.08 (0.96;1.21)	0.99 (0.83;1.17)	1.27 (1.10;1.46)	1.38 (1.00;1.83)	0.91 (0.71;1.16)	1.06 (0.74;1.46)	1.14 (1.08;1.2)	1.2 (1.11;1.29)
2016	1.16 (1.07;1.25)	1.26 (1.13;1.4)	1.01 (0.89;1.14)	0.92 (0.76;1.1)	1.25 (1.07;1.44)	1.23 (0.87;1.70)	0.88 (0.69;1.11)	1.03 (0.7;1.42)	1.1 (1.04;1.16)	1.14 (1.05;1.23)
2017	1.14 (1.05;1.24)	1.32 (1.18;1.46)	1.10 (0.98;1.24)	1.05 (0.88;1.25)	1.36 (1.17;1.59)	1.15 (0.81;1.58)	0.88 (0.67;1.12)	1.13 (0.78;1.56)	1.13 (1.07;1.19)	1.19 (1.1;1.28)
2018	1.12 (1.03;1.22)	1.27 (1.13;1.41)	0.99 (0.88;1.12)	0.98 (0.81;1.17)	1.33 (1.13;1.56)	1.28 (0.89;1.74)	1.07 (0.83;1.35)	0.96 (0.64;1.38)	1.08 (1.02;1.15)	1.16 (1.07;1.25)
